# Multiple truncated isoforms of MAVS prevent its spontaneous aggregation in antiviral innate immune signalling

**DOI:** 10.1038/ncomms15676

**Published:** 2017-06-13

**Authors:** Nan Qi, Yuheng Shi, Rui Zhang, Wenting Zhu, Bofeng Yuan, Xiaoyan Li, Changwan Wang, Xuewu Zhang, Fajian Hou

**Affiliations:** 1 State Key Laboratory of Cell Biology, Innovation Center for Cell Signaling Network, CAS Center for Excellence in Molecular Cell Science, Shanghai Institute of Biochemistry and Cell Biology, Chinese Academy of Sciences; University of Chinese Academy of Sciences, 320 Yueyang Road, Shanghai 200031, China; 2 Department of Pharmacology, University of Texas Southwestern Medical Center, Dallas, Texas 75039, USA

## Abstract

In response to virus infection, RIG-I-like receptors (RLRs) sense virus RNA and induce MAVS to form prion-like aggregates to further propagate antiviral signalling. Although monomeric MAVS recombinant protein can assemble into prion-like filaments spontaneously *in vitro*, endogenous MAVS in cells is prevented from aggregation until viral infection. The mechanism preventing cellular MAVS from spontaneous aggregation is unclear. Here we show that multiple N-terminal truncated isoforms of MAVS are essential in preventing full-length MAVS from spontaneous aggregation through transmembrane domain-mediated homotypic interaction. Without these shorter isoforms, full-length MAVS is prone to spontaneous aggregation and Nix-mediated mitophagic degradation. In the absence of N-terminally truncated forms, blocking Nix-mediated mitophagy stabilizes full-length MAVS, which aggregates spontaneously and induces the subsequent expression of type I interferon and other proinflammatory cytokines. Our data thus uncover an important mechanism preventing spontaneous aggregation of endogenous MAVS to avoid accidental activation of antiviral innate immune signalling.

## Abstract (web summary)

MAVS is an essential component of the pathogen-sensing machinery, and functions by forming prion-like filaments. Here the authors show that alternatively translated forms of truncated endogenous MAVS can prevent spontaneous aggregation and degradation in cells to sustain MAVS-mediated immune signalling.

Innate immune response has a central function against viral infection^1^,^2^. On virus infection, viral nucleic acids can serve as pathogen-associated molecular patterns (PAMP) and are detected by pattern recognition receptors (PRR) in host cells. RIG-I-like receptors (RLRs) are a major PRR in sensing intracellular viral RNA and mediating antiviral response^3^,^4^. RLR pathways initiate a signalling cascade to activate transcriptional factors IRF3 and NF-κB and results in the expression of type I interferon (IFN), proinflammatory cytokines and subsequent IFN stimulated genes (ISGs) to eventually restrict virus proliferation. Signalling leading to type I IFN production is tightly controlled to prevent excessive and detrimental inflammation by numerous negative feedback mechanisms^5^,^6^.

Mitochondrial antiviral-signalling protein (MAVS, also known as IPS-1, VISA and CARDIF) is a crucial adaptor in the RIG-I pathway^7^,^8^,^9^,^10^. RIG-I binds to viral RNA and undergoes a conformational change to release its N-terminal tandem caspase activation and recruitment domains (2CARD)^11^,^12^,^13^. RIG-I 2CARD then interacts with the MAVS CARD domain and induces it to form prion-like filaments to further transduce antiviral signals^14^,^15^,^16^,^17^. Prion-like filament formation of MAVS is crucial for its activation, but the regulatory mechanism is unclear. MAVS is composed of an N-terminal CARD domain, three active regions (Region I/II/III) in the middle and a C-terminal transmembrane domain (TM)^18^. MAVS CARD domain alone is sufficient to form prion-like aggregates. The three active regions provide binding sites for MAVS to recruit downstream signalling molecules, and posttranslational phosphorylation of MAVS Region III has an essential function for its downstream signalling^19^. The TM domain tethers MAVS to the mitochondria, which is critical for its antiviral function but the underlying mechanism is unclear^8^. Both recombinant MAVS-ΔTM and isolated CARD can form prion-like aggregates spontaneously *in vitro*, but endogenous MAVS in cells remains monomeric and quiescent until virus infection^14^, indicating the involvement of a critical mechanism that prevents endogenous MAVS from spontaneous aggregation.

Overexpressed MAVS is reported to induce reactive oxygen species (ROS) and autophagy, which might regulate type I interferon signalling^20^. Autophagy-defective cells are shown to exhibit enhanced RLR signalling upon stimulation, suggesting a negative function of autophagy in the RIG-I pathway^21^. However, how autophagy or mitophagy regulates endogenous MAVS in the absence of stimulation has not been defined. Autophagy mediates metabolic processes that are important for maintaining cellular homoeostasis by degrading protein aggregates, damaged organelles or intracellular pathogens^22^. Non-selective autophagy occurs during cellular starvation when the cytosol is recycled to provide nutrition, and selective autophagy is induced when specific substrates are targeted for autophagic degradation. Selective autophagy is an important mechanism to eliminate cytosol-invading viruses, parasites and bacteria. In addition, mitophagy is also a typical selective process when unwanted or damaged mitochondria are cleared by autophagy pathway^23^. Mitochondrial kinase PINK1 and E3 ubiquitin ligase Parkin mediate mitophagy by promoting ubiquitination of outer mitochondrial membranous proteins^24^. The signals for protein degradation, such as ubiquitin, can then be recognized by a variety of adaptor proteins or cargo receptors, including p62 (SQSTM1), Optineurin, NDP52, Tollip, TAX1BP1 and NBR1, which bridge ATG8 family proteins on the autophagosomal membranes to ubiquitinated cargoes^25^,^26^. In mammalian cells, mitochondrial protein Nix can mediate PINK1-Parkin-independent mitophagy by directly binding to ATG8 family members on the phagophore^27^,^28^,^29^.

Here we identify multiple N-terminally truncated isoforms of MAVS, which lack a MAVS CARD domain. These truncated isoforms and full-length MAVS can associate with each other through homotypic interaction mediated by their TM domains. We further demonstrate that these truncated isoforms have an essential function in keeping full-length MAVS from spontaneous aggregation. In the absence of N-terminally truncated isoforms, full-length MAVS forms spontaneous aggregates, and is cleared by Nix-mediated mitophagic degradation. Therefore, our studies uncover a mechanism to prevent MAVS spontaneous aggregation in cells, which avoids the misfiring of innate immune response for potential detrimental inflammation, and it might also provide a general mechanism in preventing spontaneous aggregation of prion-like proteins in cellular signalling.

## Results

### Identification of N-terminally truncated isoforms of MAVS

**Figure 1:**
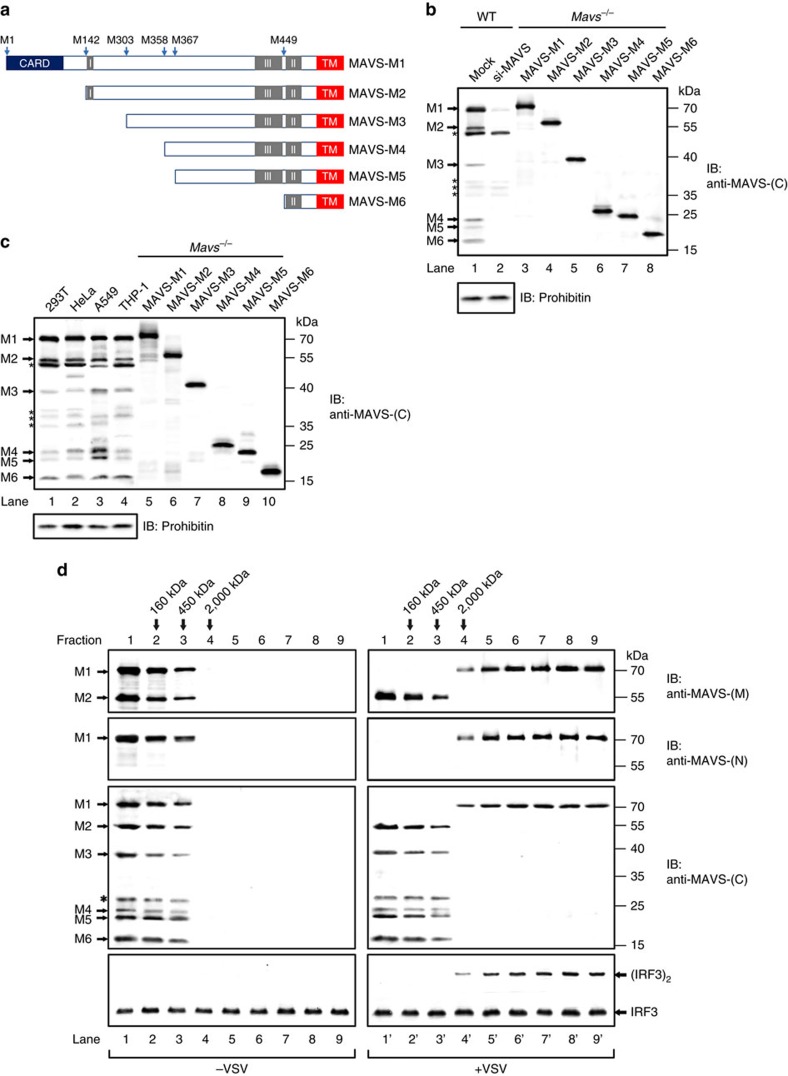
N-terminally truncated isoforms of MAVS were separated from full-length MAVS upon virus infection. (**a**) A diagram illustrating that six MAVS isoforms, including MAVS- M1/2/3/4/5/6, are translated from the polycistronic *Mavs* transcript. (**b**) Immunoblotting showing that six MAVS isoforms are expressed in HEK293T cells. P5 fractions containing mitochondria were obtained from HEK293T cells treated with or without siRNA oligoes targeting *Mavs* (si-MAVS) and subjected for immunoblotting (Lane 1, 2). Lane 3–8, HEK293T *Mavs*^*−/−*^ cells were transfected with constructs expressing Flag-tagged MAVS-M1/M2/M3/M4/M5/M6, respectively. Whole cell lysates were then prepared at thirty-six hours post transfection and used for SDS-PAGE and immunoblotting, to indicate the migration position of the ectopically expressed MAVS isoforms in Lane 1 and 2. Mitochondrial outer membrane protein Prohibitin was immunoblotted as an internal control. Endogenous MAVS isoforms were indicated as arrows. Non-specific bands were labelled with asterisks. The original full blot can be found in Supplementary Fig. 11a. (**c**) Multiple isoforms of MAVS were detected in various human cell lines. P5 fractions from HEK293T, HeLa, A549 and THP-1 were obtained and subjected to immunoblotting with anti-MAVS-(C) antibody (Lane 1–4). Prohibitin was immunoblotted as an internal control. As described in (**b** lane 3–8), Flag-tagged MAVS isoforms were immunoblotted to indicate the migration position of MAVS truncated forms (Lane 5–10). Endogenous MAVS isoforms were indicated as arrows. Non-specific bands were labelled with asterisks. See also Supplementary Fig. 1b. The original full blot can be found in Supplementary Fig. 11b. (**d**) HEK293T cells infected with or without vesicular stomatitis virus (VSV) for 12 h were collected and subjected to subcellular fractionation. P5 fractions containing mitochondria were collected and solubilized with n-Dodecyl-β-D-maltoside (DDM) before sucrose gradient centrifugation. Nine fractions from top to the bottom were taken and analysed by immunoblotting with anti-MAVS-(C), anti-MAVS-(M) and anti-MAVS-(N) antibodies. MAVS activities in stimulating IRF3 dimerization were also examined. Arrows indicate the peaks of standard proteins (160 and 450 kDa) and blue dextran 2,000 (2,000 kDa) used as molecular weight markers. Asterisk indicates a non-specific band. The original full blot can be found in Supplementary Fig. 11c.

In addition to full-length MAVS, a N-terminally truncated isoform of MAVS ∼50 kDa was observed in many reports^7^,^14^,^30^. The truncated isoform comigrates with full-length MAVS on the sucrose gradient centrifugation in the absence of stimulation. On virus infection, the truncated isoform is separated from full-length MAVS when the latter forms functional prion-like aggregates^14^. Both the truncated form and full-length MAVS were produced from the same bicistronic *Mavs* transcript^31^,^32^. The key feature of bicistronic mRNA is that protein translation could initiate not only from the very N-terminal methionine but also from internal methionine alternately, thus generating truncated protein products. MAVS harbours six methionines (M1/142/303/358/367/449), which could potentially result in six isoforms by the so-called polycistronic mechanism, namely MAVS-M1/2/3/4/5/6 (Fig. 1a). However, only two isoforms of MAVS, including MAVS-M1 and M2, have been reported up to date. To determine if there are other truncated isoforms of MAVS as predicted, three antibodies named anti-MAVS-(N), anti-MAVS-(M) and anti-MAVS-(C) were raised against MAVS N-terminal CARD domain aa-1–100, middle region aa-301–460 and C-terminal region aa-460–510 respectively. In addition, putative MAVS-M1/2/3/4/5/6 were cloned and expressed in HEK293T *Mavs*^*−/−*^ cells with Flag tag. Among the three antibodies, only anti-MAVS-(C) can recognize all of these putative MAVS isoforms or fragments (Supplementary Fig. 1a).

Using anti-MAVS-(C), we detected six endogenous MAVS isoforms in HEK293T cells, whose migration positions correlated with their Flag-tagged counterparts respectively (Fig. 1b). Notably, si-MAVS treatment knocked-down the expression of these isoforms specifically but not some non-specific bands, indicating that the six MAVS isoforms are produced from the same transcript. These MAVS isoforms but MAVS-M1 were not detected by anti-MAVS-(N), indicating all of them are N-terminally truncated isoforms. We also examined a variety of human cell lines and found that multiple N-terminally truncated isoforms of MAVS were expressed at various levels (Fig. 1c and Supplementary Fig. 1b). To determine if these MAVS isoforms come from different transcripts, we performed reverse-transcribed PCR (RT-PCR) for *Mavs* transcripts from various cell lines using primers from both 5′ and 3′ untranslational region (UTR), which generated a single band, demonstrating that multiple MAVS isoforms are translated from the same *Mavs* transcript by the polycistronic mechanism rather than alternative splicing (Supplementary Fig. 1c). Furthermore, these N-terminally truncated isoforms comigrated with full-length MAVS on the sucrose gradient centrifugation in the absence of VSV infection and were separated from full-length MAVS aggregates upon VSV infection (Fig. 1d). These N-terminally truncated isoforms are localized to mitochondria regardless of viral infection (Supplementary Fig. 1d). We next investigated the function of these N-terminally truncated isoforms of MAVS.

### Truncated forms of MAVS inhibit full-length MAVS aggregation

**Figure 2:**
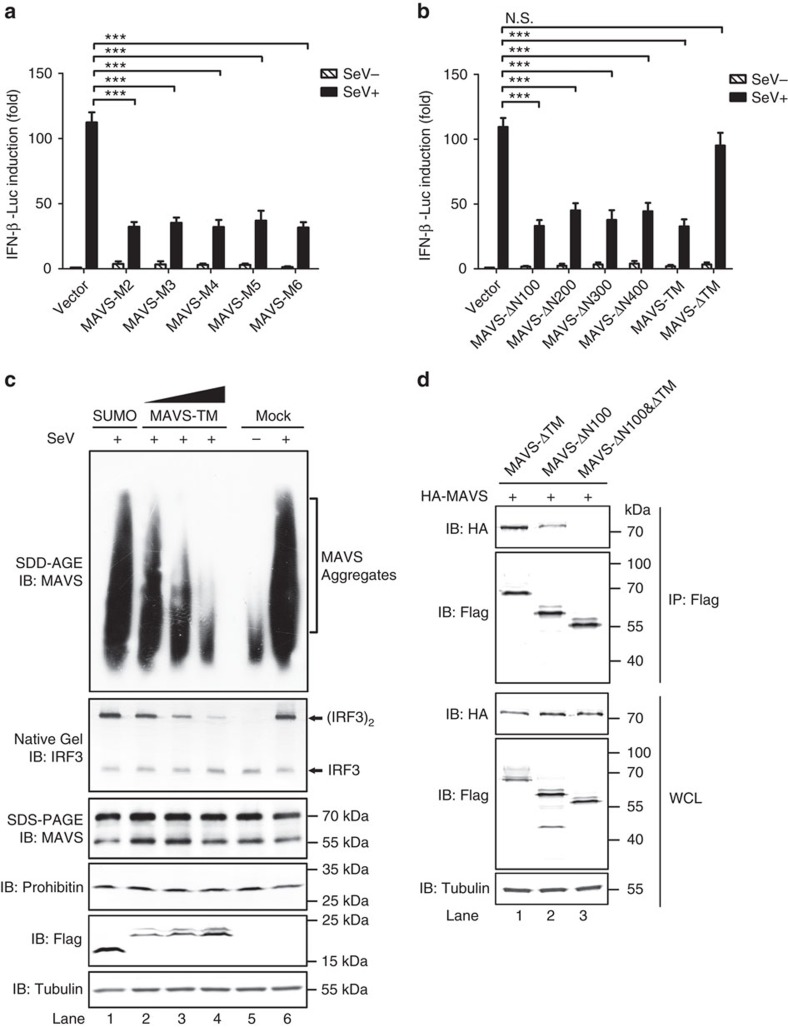
N-terminally truncated forms of MAVS prevent the aggregation of full-length MAVS. (**a**) pcDNA3-flag-MAVS-M2/M3/M4/M5/M6 or (**b**) constructs expressing a series of MAVS truncations as indicated were transfected into HEK293T cells together with IFN-luciferase reporter. Cells were infected with Sendai virus 24 h after transfection. IFN induction was measured 12 h post infection by luciferase reporter assay. See also Supplementary Fig. 2a,c. All data are presented as the mean values based on three independent experiments, and error bars indicate s.d. *P* values were determined by unpaired two-tailed Student’s *t*-test. ***P<0.001. N.S. indicates no statistically significant difference. (**c**) HEK293T cells were transfected with pcDNA3-flag-sumo, pcDNA3-flag-sumo-MAVS-TM or not. The cells were infected with Sendai virus twenty-four hours after transfection. Cells were collected 12 h post virus infection, which were subjected to subcellular fractionation to obtain P5 and S5 fractions. P5 fractions were used to examine MAVS aggregation and activity in inducing IRF3 dimerization. The original full blot can be found in Supplementary Fig. 12a. (**d**) pcDNA3-HA-MAVS was transfected into HEK293T cells together with pcDNA3-flag-MAVS-ΔTM, pcDNA3-flag-MAVS-ΔN100 or pcDNA3-flag-MAVS-ΔN100&ΔTM. Thirty-six hours after transfection, cells were collected and subjected to immunoprecipitation.

It was shown that a MAVS mutant, which lacks of amino acids 10–77, functioned as a dominant-negative form on virus infection^8^. Consistently, recent reports showed that the truncated isoform MAVS-M2 ∼50 kDa confers an inhibitory effect on IFN production^31^,^32^. In line with this, we found that expression of all N-terminally truncated MAVS isoforms dampened IFN production upon viral stimulation (Fig. 2a). To investigate the mechanism underlying this inhibitory effect, a series of MAVS deletions was generated (Supplementary Fig. 2b). Strikingly, all deletions including MAVS-TM, which contained the C-terminal 30 amino acids encoding MAVS TM domain fused to an N-terminal SUMO moiety, conferred a dominant-negative effect on endogenous MAVS (Fig. 2b). In contrast, MAVS-ΔTM could not inhibit the IFN production on virus infection, suggesting that TM domain is indispensable for the inhibitory effect observed. MAVS TM domain does not harbour any active region or Regions I/II/III, arguing against the possibility that it might compete with full-length MAVS in interacting with downstream signalling molecules. We hypothesized that MAVS-TM might prevent full-length MAVS forming prion-like aggregates. Indeed, aggregation of endogenous MAVS upon virus infection was largely inhibited when MAVS-TM is expressed transiently (Fig. 2c). As expected, IRF3 dimerization was also severely impaired. Consistently, expression of various MAVS N-terminally truncated isoforms also conferred negative effects on endogenous MAVS aggregation (Supplementary Fig. 2d). We next sought for the biochemical mechanism underlying the inhibitory effect of MAVS-TM on MAVS filament formation.

In light of that MAVS CARD domain could mediate a homotypic interaction responsible for its prion-like filament formation^14^,^15^,^33^,^34^, we tested whether MAVS TM domain could also mediate a homotypic interaction. Various MAVS deletions were tagged with different epitopes and immunoprecipitations (IP) were then performed. Full-length MAVS could be pulled-downed by MAVS-ΔN100 and MAVS-ΔTM but not by MAVS-ΔN100&ΔTM, suggesting that CARD and TM could independently mediate homotypic interaction (Fig. 2d).

### MAVS TM mediates a specific homotypic interaction

**Figure 3:**
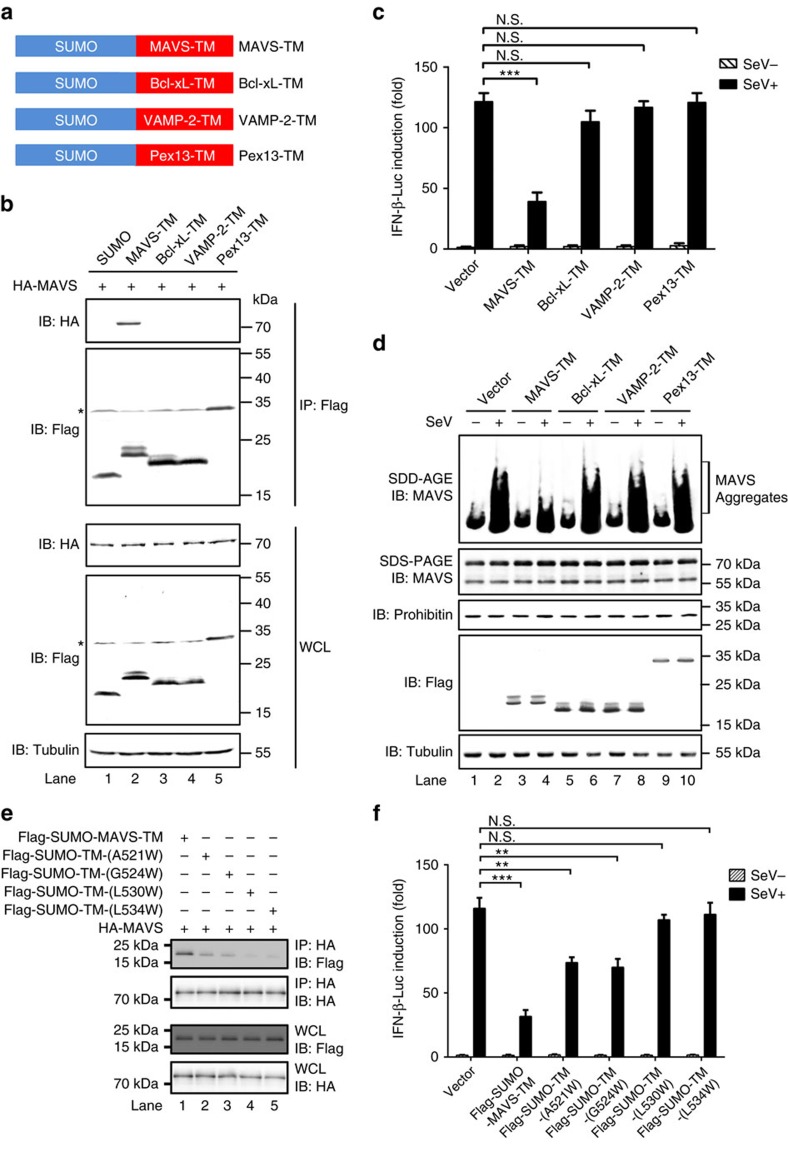
MAVS-TM inhibits aggregation of endogenous MAVS through a specific homotypic interaction. (**a**) A diagram showing SUMO tagged MAVS-TM, Bcl-xL-TM, VAMP-2-TM, Pex13-TM. (**b**) pcDNA3-HA-MAVS was transfected into HEK293T cells together with pcDNA3-flag-sumo, pcDNA3-flag-sumo-MAVS-TM, pcDNA3-flag-sumo-Bcl-xL-TM, pcDNA3-flag-sumo-VAMP-2-TM, or pcDNA3-flag-sumo-Pex13-TM. Thirty-six hours post transfection, cells were collected and subjected to immunoprecipitation. The original full blot can be found in Supplementary Fig. 12b. (**c**) Empty vector or plasmids encoding various TMs as described in (**b**) were transfected into HEK293T cells together with IFN-luciferase reporter. Cells were infected with Sendai virus twenty-four hours after transfection, and IFN induction were measured twelve hours post infection by luciferase reporter assay. All data are presented as the mean values based on three independent experiments, and error bars indicate s.d. *P* values were determined by unpaired two-tailed Student’s *t*-test. ***P<0.001. N.S. indicates no statistically significant difference. (**d**) Experiments were performed as described in (**c**) except that IFN-luciferase reporter was omitted. Cells were collected and P5 fractions were isolated to examine MAVS aggregation. Whole cell lysates were subjected to SDS–PAGE and immunoblotting. (**e**) pcDNA3-HA-MAVS was transfected into HEK293T cells together with pcDNA3-flag-sumo-MAVS-TM, pcDNA3-flag-sumo-TM-(A521W), pcDNA3-flag-sumo-TM-(G524W), pcDNA3-flag-sumo-TM-(L530W) or pcDNA3-flag-sumo-TM-(L534W). Thirty-six hours post transfection, cells were collected and subjected to immunoprecipitation with HA beads and following immunoblotting. (**f**) Empty vector or constructs encoding MAVS-TM and various mutants as described in (**e**) were transfected into HEK293T cells together with IFN-luciferase reporter. Cells were infected with Sendai virus twenty-four hours after transfection, and IFN induction were measured twelve hours post infection by luciferase reporter assay. All data are presented as the mean values based on three independent experiments, and error bars indicate s.d. *P* values were determined by unpaired two-tailed Student’s *t*-test. **P<0.01, ***P<0.001. N.S. indicates no statistically significant difference.

We further tested TMs from other membrane proteins for probable association with MAVS TM. These TMs, including Bcl-xL-TM, VAMP-2-TM and Pex13-TM, are peptide sequences that target these proteins to specific subcellular compartments (Fig. 3a)^8^,^30^. Bcl-xL is another mitochondrial outer membrane protein. VAMP-2 is primarily an ER membrane protein and Pex13 is localized on peroxisomal membrane. As expected, flag-tagged MAVS-TM could pull-down HA-tagged full-length MAVS, confirming the homotypic interaction of MAVS TMs (Fig. 3b). In contrast, full-length MAVS could not bind to flag-tagged Bcl-xL-TM, VAMP-2-TM or Pex13-TM, highlighting the specificity of MAVS-TM-mediated homotypic interaction. Strikingly, Bcl-xL-TM, VAMP-2-TM or Pex13-TM could not inhibit MAVS activity in inducing IFN production following virus infection (Fig. 3c), nor was MAVS aggregation affected (Fig. 3d). These results highlight the importance of the specific homotypic interaction mediated by MAVS TM in the inhibition of MAVS truncated forms to IFN induction. Furthermore, we made two chimeric mutants MAVS-ΔN141 by replacing MAVS TM with Bcl-xL-TM or VAMP-2-TM. Compared to MAVS-ΔN141, these chimeric mutants MAVS-ΔN141-(Bcl-xL-TM) and MAVS-ΔN141-(VAMP-2-TM) lost their inhibitory effects on IFN production and MAVS aggregation upon virus infection (Supplementary Fig. 3a,b), though they contain active regions for interaction with downstream effectors. This result further demonstrated that N-terminally truncated forms of MAVS play dominant-negative roles through the TM domain but not other regions.

We next identified four highly conserved amino acids in MAVS TMs across various species (Supplementary Fig. 3c). By mutagenesis on these conserved residues, we found that residues Leu530 and Leu534 are critical for MAVS-TM-mediated homotypic interaction and the resultant dominant-negative effect on IFN production; while the A521W and G524W mutants demonstrated impaired homotypic interaction and partially inhibit IFN production (Fig. 3e,f). Collectively, our data suggested that MAVS TM is both essential and sufficient for the truncated forms to prevent the aggregation of full-length MAVS through homotypic interaction.

### Absence of MAVS truncated isoforms leads to its activation

**Figure 4:**
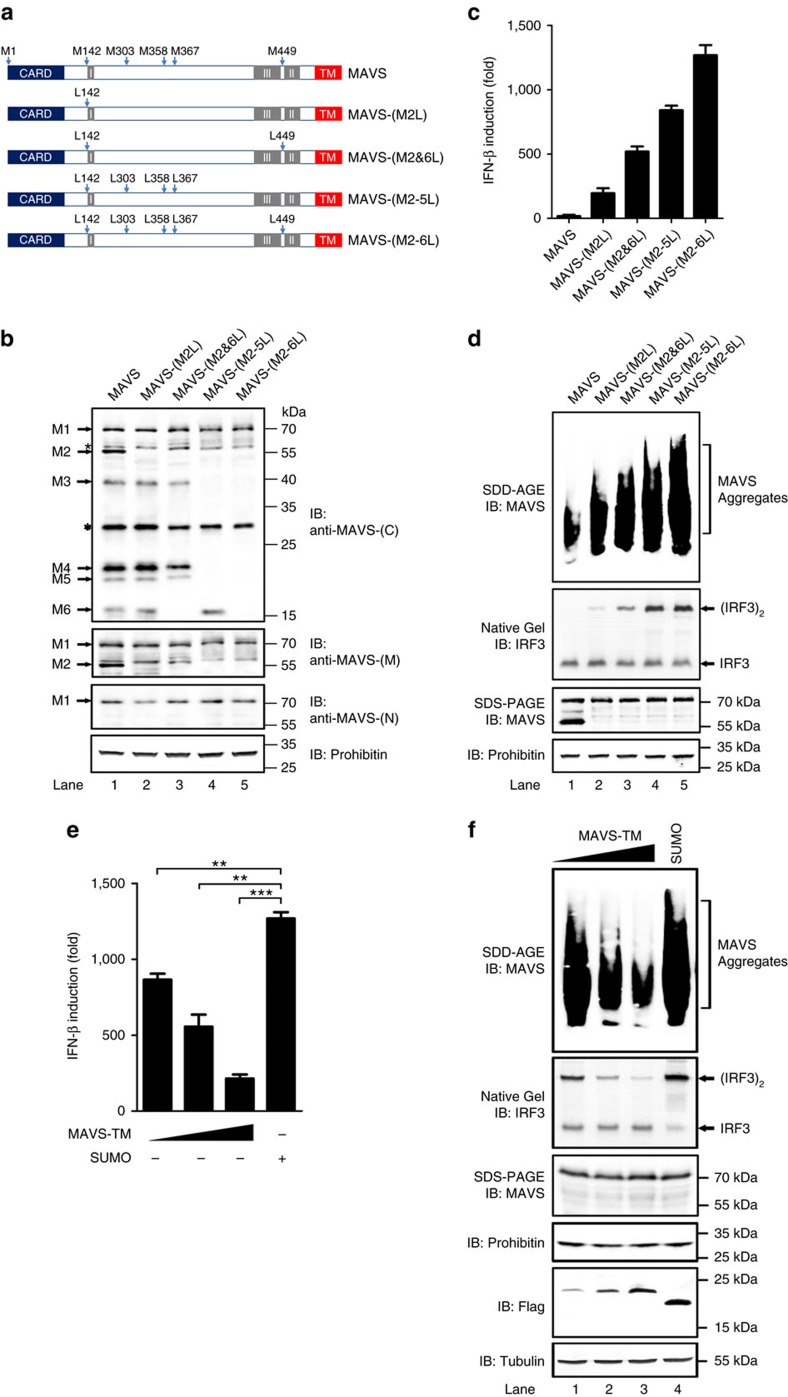
Deletion of N-terminally truncated isoforms triggers aggregation and activation of full-length MAVS. (**a**) A diagram showing various MAVS mutants with methionine replaced by leucine, including MAVS-(M2L), MAVS-(M2&6L), MAVS-(M2-5L), MAVS-(M2-6L), which abolish the production of corresponding N-terminally truncated forms. (**b**) Plasmids encoding MAVS, MAVS-(M2L), MAVS-(M2&6L), MAVS-(M2-5L), MAVS-(M2-6L) were transfected into HEK293T *Mavs*^−/−^ cells. Cells were collected 36 h post transfection. P5 fractions were isolated and used to analyse various MAVS isoforms by immunoblotting with anti-MAVS-(C), anti-MAVS-(M) and anti-MAVS-(N) antibodies. The original full blot can be found in Supplementary Fig. 12c. (**c**,**d**) Transfection was performed as described in **b**. After cells were collected, RNA was extracted and qPCR was performed to measure IFN induction (**c**). P5 fractions were isolated to examine MAVS aggregation and activity in inducing IRF3 dimerization *in vitro* (**d**). (**e**,**f**) Plasmid encoding MAVS-(M2-6L) was transfected into HEK293T *Mavs*^−/−^ cells, together with pcDNA3-flag-sumo or increasing amounts of pcDNA3-flag-sumo-MAVS-TM. As described in (**c**), IFN induction was measured by qPCR (**e**). P5 fractions were used to examine MAVS aggregation and activity in inducing IRF3 dimerization *in vitro* (**f**). Cell lysates were subjected to SDS-PAGE and immunoblotting. All data are presented as the mean values based on three independent experiments, and error bars indicate s.d. *P* values were determined by unpaired two-tailed Student’s *t*-test. **P<0.01 and ***P<0.001. N.S. indicates no statistically significant difference.

We next investigated the physiological function of N-terminally truncated isoforms of MAVS in cells. As these truncated isoforms are translated by alternative initiation from internal methionine, mutation of an internal methionine would abrogate the production of corresponding truncated isoform (Fig. 4a). Various forms of MAVS were transiently expressed in HEK293T *Mavs*^*−/−*^ cells and immunoblotting was performed (Fig. 4b). Indeed, N-terminally truncated isoforms were expressed with wild type full-length MAVS and specific truncated isoforms disappeared in corresponding mutants. These results confirmed that N-terminally truncated isoforms are generated from the polycistronic *Mavs* transcript, and we concluded that six isoforms of MAVS could be translated from the polycistronic *Mavs* transcript.

Full-length MAVS plays an essential role in antiviral signalling. We then asked whether N-terminally truncated isoforms could be involved in antiviral signalling. MAVS-M2 did not form prion-like aggregates and could be separated very well from full-length MAVS biochemically following virus infection^14^. On the other hand, prion-like aggregates formed by full-length MAVS are both sufficient and essential to transduce antiviral signal to downstream effectors, suggesting that MAVS-M2 might not be required for antiviral signalling *per se*. To investigate the function of MAVS truncated isoforms, we evaluated the effect of mutations disrupting the expression of these truncated isoforms. Wild type MAVS was expressed in HEK293T *Mavs*^*−/−*^ cells at a near-physiological level to avoid spontaneous activation. Strikingly, though expressed at levels comparable to wild type MAVS, various MAVS mutants showed robust activities in stimulating IFN induction (Fig. 4c). The activities of MAVS mutants correlated with the disappearance of their respective truncated isoforms. To investigate the mechanisms underlying the spontaneous activation of these MAVS mutants, we examined the aggregation of these various forms of MAVS. Remarkably, MAVS mutants, but not wild type MAVS, form prion-like aggregates in the absence of virus infection (Fig. 4d). The mutant MAVS-(M2-6L), which is deprived of all truncated isoforms, showed the strongest activity in stimulating IFN and most pronounced aggregation. Notably, IFN induction by MAVS-(M2-6L) could be blocked by the coexpression of MAVS-TM in a dosage-dependent manner (Fig. 4e), which is consistent with the inhibitory effect of ectopically expressed MAVS-TM on endogenous MAVS through homotypic interaction. In support of this, spontaneous aggregation of MAVS-(M2-6L) was disrupted by transient expression of MAVS-TM (Fig. 4f). These results promoted us to hypothesize that N-terminally truncated isoforms of endogenous MAVS could play an essential role in keeping full-length MAVS from spontaneous aggregation and activation.

### Absence of truncated isoforms leads to MAVS degradation

**Figure 5:**
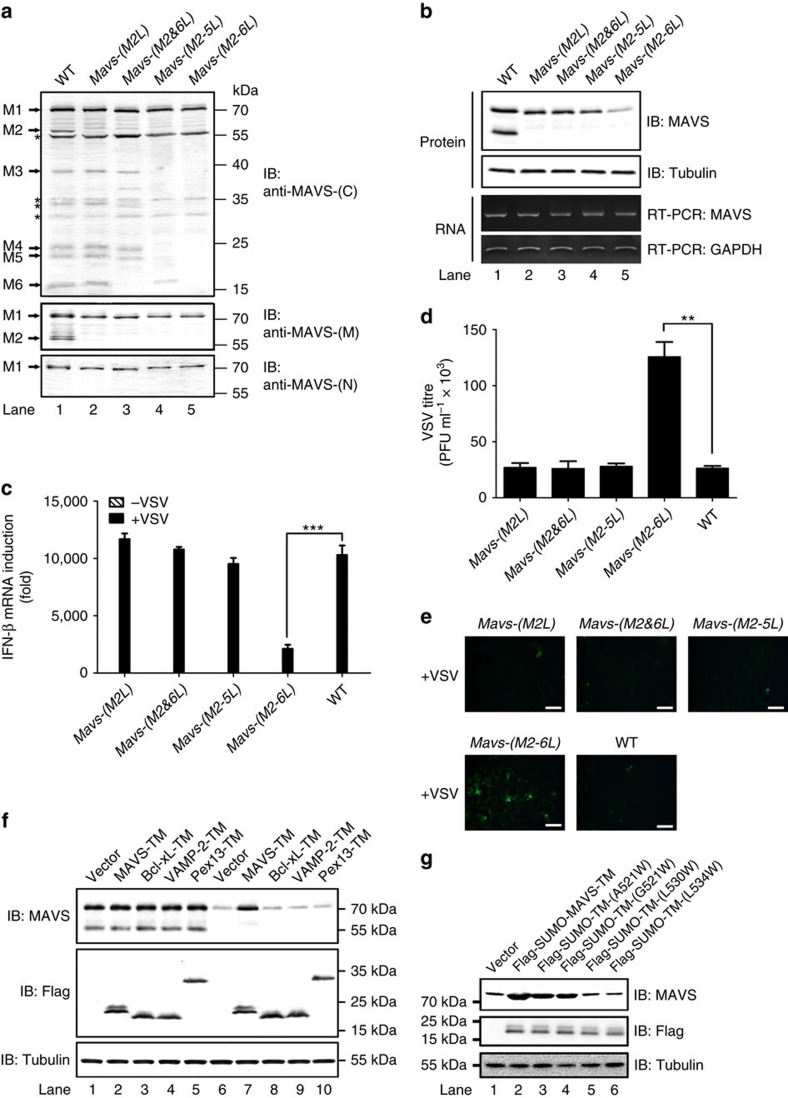
Endogenous MAVS is degraded in the absence of its N-terminally truncated isoforms. (**a**) HEK293T WT, *Mavs-(M2L)*, *Mavs-(M2&6L)*, *Mavs-(M2-5L)* and *Mavs-(M2-6L)* cells were subjected to subcellular fractionation to obtain P5 fractions. Various MAVS isoforms were detected by immunoblotting with anti-MAVS-(C), anti-MAVS-(M) and anti-MAVS-(N) antibodies. See also Supplementary Fig. 4a. The original full blot can be found in Supplementary Fig. 13a. (**b**) Whole cell lysates of HEK293T WT, *Mavs-(M2L)*, *Mavs-(M2&6L)*, *Mavs-(M2-5L)* and *Mavs-(M2-6L)* cells was obtained to determine endogenous MAVS protein level by immunoblotting. Tubulin was immunoblotted as a loading control. RT-PCR was performed to measure mRNA level of various *Mavs* gene. GAPDH was analysed as an internal control. (**c**–**e**) HEK293T WT, *Mavs-(M2L)*, *Mavs-(M2&6L)*, *Mavs-(M2-5L)* and *Mavs-(M2-6L)* cells were infected with VSV (MOI=1). Twelve hours post infection, RNA was extracted and qPCR was performed to measure IFN induction (**c**). VSV titres were quantitated by plaque assay (**d**). Fluorescent images were taken to examine VSV proliferation eight hours after infection (**e**). Scale bar represents 10 micrometres. All data are presented as the mean values based on three independent experiments, and error bars indicate s.d. *P* values were determined by unpaired two-tailed Student’s *t*-test. **P<0.01, ***P<0.001. (**f**) HEK293T WT (lane 1–5) and *Mavs-(M2-6L)* (lane 6–10) cells were transfected with pcDNA3-flag-sumo-MAVS-TM, pcDNA3-flag-sumo-Bcl-xL-TM, pcDNA3-flag-sumo-VAMP-2-TM, pcDNA3-flag-sumo-Pex13-TM or empty vector. After thirty-six hours, cells were subjected to the second transfection. Cells were collected 36 h post the second transfection. Whole cell lysates were obtained to determine the endogenous MAVS protein level by immunoblotting. (**g**) Plasmids expressing SUMO, MAVS-TM and its mutants as indicated were transfected into HEK293T *Mavs-(M2-6L)* cells. Transfection was performed as described in **f**. Whole cell lysates were obtained to determine the endogenous MAVS protein level by immunoblotting.

To determine the physiological function of truncated isoforms of endogenous MAVS, we edited *Mavs* gene in the genome of HEK293T cells by the transcription activator-like effector nucleases (TALEN) technique. All of the internal methionine codon ATGs was changed to CTGs so that the translation of corresponding truncated isoforms from polycistronic *Mavs* transcript was blocked. As a result, full-length MAVS could be expressed except that its internal methionine residues were mutated to leucine. We obtained four alleles, including *Mavs-(M2L), Mavs-(M2&6L), Mavs-(M2-5L)* and *Mavs-(M2-6L)*. MAVS truncated isoforms were not detected in their respective mutant alleles as expected (Fig. 5a and Supplementary Fig. 4a). To our surprise, MAVS-(M2-6L), from the allele that was devoid of any N-terminally truncated isoforms, was expressed at a very low level, in contrast to wild type MAVS or other mutants (Fig. 5b). Low-protein level of MAVS-(M2-6L) was not due to transcriptional regulation, as comparable amounts of mRNAs were detected in all four mutant alleles as well as wild type cells. Accordingly, loss of MAVS-(M2-6L) resulted in compromised IFN production and antiviral effect on viral proliferation in *Mavs-(M2-6L)* cells upon VSV infection (Fig. 5c–e). One explanation is that MAVS-(M2-6L) is degraded once N-terminally truncated isoforms are absent. Indeed, transient expression of MAVS-TM or N-terminally truncated isoforms in *Mavs-(M2-6L)* cells could stabilize full-length MAVS (Fig. 5f and Supplementary Fig. 4b). On the contrary, mutants MAVS-TM-(L530W) and MAVS-TM-(L534W) could not restore MAVS-(M2-6L) expression (Fig. 5g), further demonstrating that the MAVS-TM-mediated homotypic interaction are critical for MAVS stabilization by its N-terminally truncated isoforms.

### Spontaneously-aggregated MAVS is degraded by autophagy

**Figure 6:**
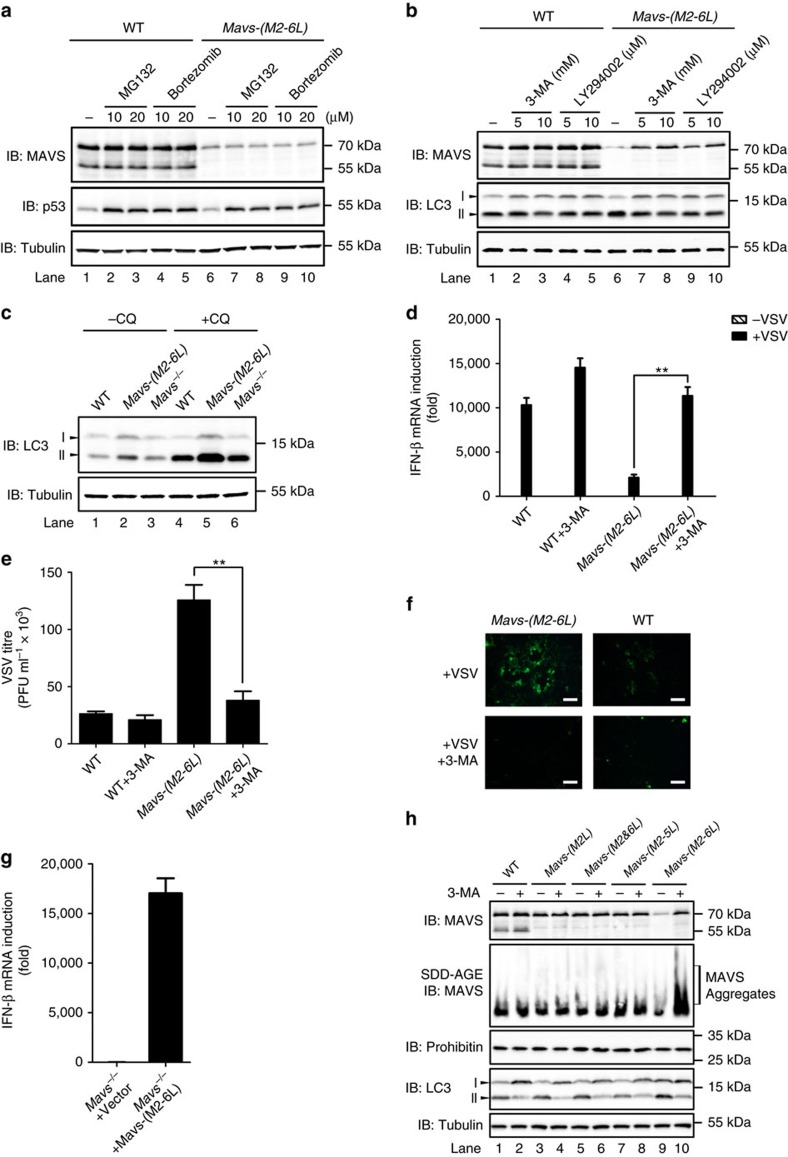
Spontaneously-aggregated MAVS in *Mavs-(M2-6L)* cells is degraded by autophagic pathway. (**a**) HEK293T WT (lane 1–5) and *Mavs-(M2-6L)* cells (lane 6–10) were treated with DMSO or proteasome inhibitors MG132 and Bortezomib as indicated for four hours. Whole cell lysates were subjected to SDS-PAGE and immunoblotting. (**b**) Treatment of HEK293T WT (lane 1–5) and *Mavs-(M2-6L)* cells (lane 6–10) with DMSO or autophagy inhibitors 3-MA and LY294002 was performed for four hours. Whole cell lysates were used for SDS-PAGE and immunoblotting. The autophagy marker LC3 was immunoblotted. The original full blot can be found in Supplementary Fig. 13b. (**c**) HEK293T WT, *Mavs*^*−/−*^ and *Mavs-(M2-6L)* cells were treated with or without 30 μM chloroquine (CQ) for four hours. Whole cell lysates were used for SDS-PAGE and immunoblotting. (**d**–**f**) HEK293T WT and *Mavs-(M2-6L)* cells were treated with or without 10 mM 3-MA for two hours before infection with VSV (MOI=1). Twelve hours post infection, RNA was extracted and qPCR was performed to measure IFN induction (**d**). VSV titres were quantitated by plaque assay (**e**). Fluorescent images were taken to examine VSV proliferation eight hours after infection (**f**). Scale bar represents 10 micrometres. All data are presented as mean values based on three independent experiments, and error bars indicate s.d. *P* values were determined by unpaired two-tailed Student’s *t*-test. **P<0.01. (**g**) HEK293T *Mavs*^*−/−*^ cells were transfected with empty vector or plasmid expressing MAVS-(M2-6L). After 24 h post transfection, cells were infected with VSV (MOI=1) for 12 h. RNA were then extracted from collected cells and qPCR was performed to measure IFN induction. See also Supplementary Fig. 5b,c. All data are presented as the mean values based on three independent experiments, and error bars indicate s.d. (**h**) HEK293T WT, *Mavs-(M2L)*, *Mavs-(M2&6L)*, *Mavs-(M2-5L)* and *Mavs-(M2-6L)* cells were untreated or treated with 10 mM 3-MA for 4 h. Whole cell lysates were prepared for immunoblotting to determine the endogenous MAVS protein level. P5 fractions were isolated to examine MAVS aggregation.

Proteasome and autophagy are two major pathways mediating degradation of proteins and organelles in cells. We investigated which pathways might be responsible for MAVS-(M2-6L) degradation. Treatment with two proteasome inhibitors MG132 and bortezomib did not trigger the accumulation of MAVS-(M2-6L) in *Mavs-(M2-6L)* cells. In contrast, p53, which is known to be degraded by proteasome pathway, was elevated in cell treated with the proteasome inhibitors (Fig. 6a)^35^,^36^. These results indicated that low levels of MAVS-(M2-6L) might not be due to proteasome-mediated degradation. We then tested 3-Methyladenine (3-MA) and LY294002, which block the autophagy pathway^22^. Remarkably, 3-MA- and LY294002- treatment resulted in dramatic increase of MAVS-(M2-6L), suggesting that MAVS-(M2-6L) is degraded by autophagy pathway (Fig. 6b). LC3-II level in *Mavs-(M2-6L)* cells was much higher than wild type HEK293T cells (Fig. 6c), suggesting that autophagy was induced in *Mavs-(M2-6L)* cells. Treatment with chloroquine, a lysosomal protease inhibitor, did not affect the elevated level of LC3-II in *Mavs-(M2-6L)* cells compared with wild type HEK293T cells, indicating that the higher level of LC3-II in *Mavs-(M2-6L)* cells was due to increased synthesis rather than the blockage of its degradation in the lysosome. These results together demonstrated that autophagy was constitutively induced in *Mavs-(M2-6L)* cells. VSV infection was reported to induce autophagy as a negative regulator for type I interferon production^37^,^38^. Indeed, upon VSV infection, LC3-II was significantly increased in both wild type and *Mavs*^*−/−*^ HEK293T cells, suggesting that autophagy induction by VSV was independent of MAVS (Supplementary Fig. 5a).

When autophagy pathway was blocked by inhibitor 3-MA in *Mavs-(M2-6L)* cells, the MAVS-(M2-6L) is accumulated and the cells restored its antiviral activities to produce IFN and inhibit viral proliferation upon VSV infection (Fig. 6d–f). On the other hand, transiently expressed MAVS-(M2-6L) in *Mavs*^−/−^ cells also exhibited robust antiviral activities regardless of 3-MA treatment (Fig. 6g and Supplementary Fig. 5b,c), indicating that its antiviral activity is independent on truncated isoforms. Moreover, we found that only MAVS-(M2-6L), whose N-terminally truncated isoforms were all deleted, could be stabilized and formed prion-like aggregates with 3-MA treatment (Fig. 6h).

### Beclin 1 and ATG5 are required for MAVS degradation

**Figure 7:**
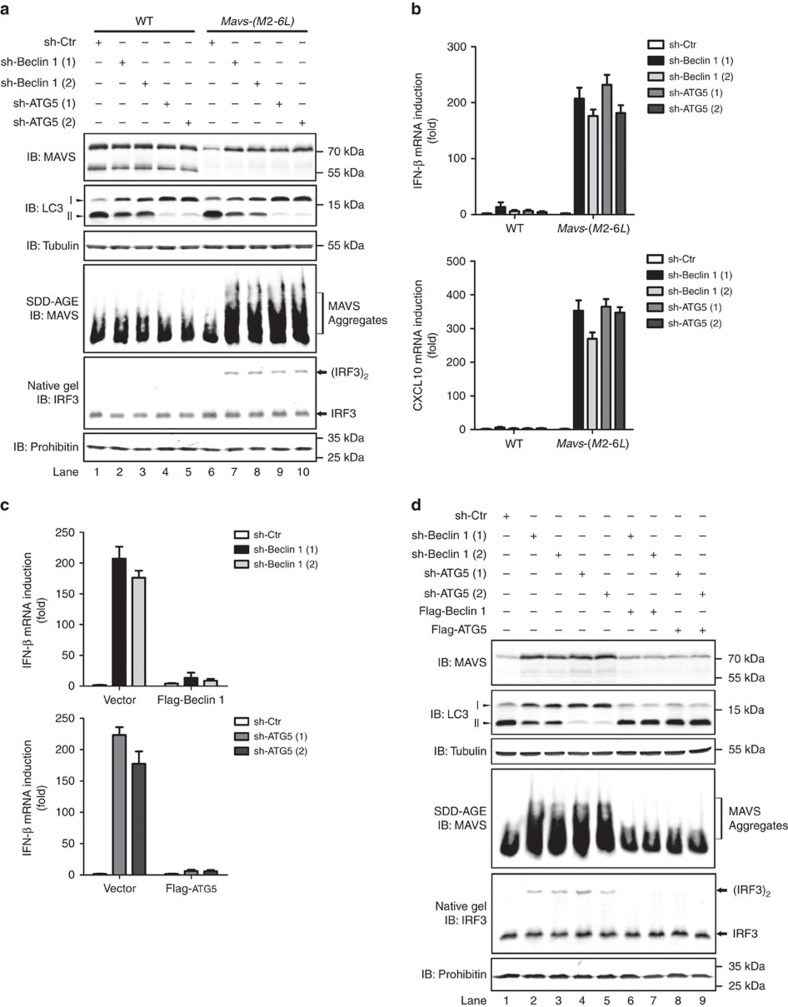
Beclin 1 and ATG5 are required for autophagic degradation of spontaneously-aggregated MAVS. (**a**,**b**) HEK293T WT and *Mavs-(M2-6L)* cells were transfected with constructs expressing sh-Beclin 1 (1 and 2) or sh-ATG5 (1 and 2). After thirty-six hours, second transfection was performed. Cells were collected 36 h post the second transfection. P5 fractions were isolated to examine MAVS aggregation and activity in inducing IRF3 dimerization *in vitro* (**a**). RNA was extracted and qPCR was performed to measure IFN induction and CXCL10 production (**b**). Whole cell lysates were also subjected to SDS-PAGE and immunoblotting. See also Supplementary Fig. 6a,b. (**c**,**d**) HEK293T *Mavs-(M2-6L)* cells were treated with shRNA as described in (**a**) in addition to the rescue vector pcDNA3-flag-Beclin 1 or pcDNA3-flag-ATG5. All data are presented as the mean values based on three independent experiments, and error bars indicate s.d. See also Supplementary Fig. 6c. The original full blot can be found in Supplementary Fig. 13c.

Beclin 1 and ATG5 are two essential components for proper autophagy-mediated degradation^39^,^40^. To determine whether the autophagy pathway are indeed responsible for MAVS-(M2-6L) instability, small hairpin RNAs (shRNA) targeting Beclin 1 or ATG5 were introduced into *Mavs-(M2-6L)* cells to block autophagy pathway. Remarkably, both sh-Beclin 1 and sh-ATG5 treatment led to marked accumulation of MAVS-(M2-6L) (Fig. 7a). These results strongly suggested that in the absence of N-terminally truncated forms, MAVS-(M2-6L) is subjected to autophagy-mediated degradation. We showed that the absence of N-terminally truncated isoforms could lead to spontaneous aggregation transiently expressed MAVS-(M2-6L) (Fig. 4d), which might be the trigger for its degradation. Therefore, we reasoned that accumulated MAVS-(M2-6L), as a result from sh-Beclin 1 or sh-ATG5 treatment, should be in the aggregated form and could activate MAVS downstream signalling. Indeed, the accumulated MAVS-(M2-6L), though at a level less than endogenous MAVS, formed prion-like aggregates as visualized on SDD-AGE and was able to stimulate IRF3 dimerization (Fig. 7a). Remarkably, the accumulated MAVS-(M2-6L) induced IFN production dramatically as well as other cytokines such as CXCL10, ISG54 and CCL5 (Fig. 7b and Supplementary Fig. 6b). Furthermore, these phenotypes could be rescued by ectopically expressing Beclin 1 or ATG5 in sh-Beclin 1- or sh-ATG5- treated cells respectively (Fig. 7c,d), confirming that the endogenous MAVS-(M2-6L) are indeed degraded through the autophagy pathway. Taken together, our results suggested that endogenous MAVS-(M2-6L) is subjected to autophagy-mediated degradation due to its spontaneous aggregation, which is a result of the absence of N-terminally truncated isoforms.

### Nix and ROS are essential for MAVS degradation

**Figure 8:**
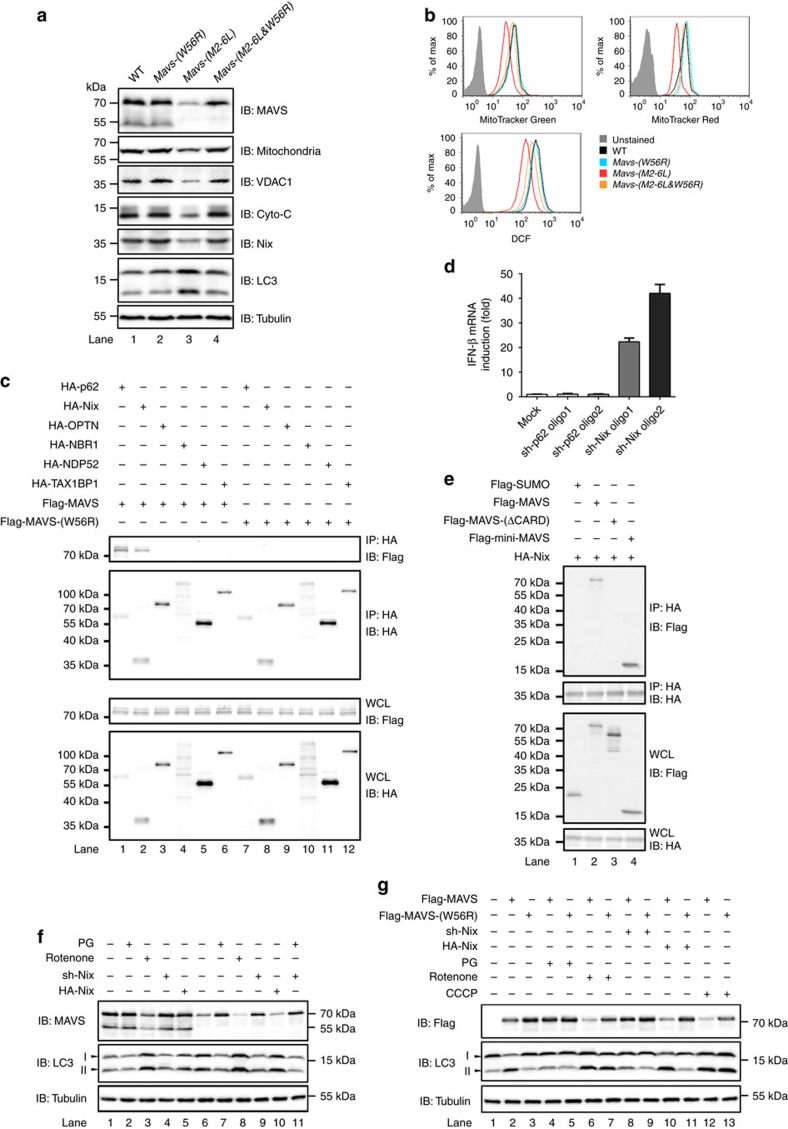
ROS and cargo receptor Nix are required for mitophagic degradation of MAVS. (**a**) Immunoblotting to show that mitochondrial turnover was induced in *Mavs-(M2-6L)* cells. Whole cell lysates of HEK293T WT, *Mavs-(W56R)*, *Mavs-(M2-6L)* and *Mavs-(M2-6L*&*W56R)* cells were prepared for SDS–PAGE. Immunoblotting was performed with indicated antibodies recognizing MAVS, VDAC1 (mitochondrial out membrane protein), cytochrome-c (Cyto-C, mitochondrial inner membrane protein), Nix (autophagy cargo receptor) and LC3 (autophagy marker). Anti-mitochondria antibody was used to determine the mitochondria number. (**b**) FACS analysis showing that mitophagy eliminated damaged mitochondria and reduced ROS production in *Mavs-(M2-6L)* cells. HEK293T WT, *Ma*vs-(W56R), Mavs-(M2-6L) and Mavs-(M2-6L&W56R) cells were stained with 200 nM Mitrotracker Green (which stains the lipid membrane of total mitochondria) and 150 nM Mitrotracker Red (which fluoresces upon oxidation in respiring mitochondria). For determination of mitochondrial ROS production, cells were stained with 2 μM DCFH-DA. After 20 min of incubation, cells were collected for FACS analysis. Histograms of FACS analysis are depicted. FACS gating strategy can be found in Supplementary Fig. 9. (**c**) pcDNA3-flag-MAVS, or pcDNA3-flag-MAVS-(W56R) were transfected into HEK293T *Mavs*^−/−^ cells together with plasmids encoding various HA-tagged autophagy cargo receptors as indicated. After 36 h of transfection, cells were collected and subjected to immunoprecipitation and immunoblotting. The original full blot can be found in Supplementary Fig. 13d. (**d**) Constructs encoding shRNAs targeting p62 or Nix were transfected into *Mavs-(M2-6L)* cell line, as described in Fig. 7a. RNA was extracted from collected cells and qPCR was performed to measure IFN induction. All data are presented as the mean values based on three independent experiments, and error bars indicate s.d. See also Supplementary Fig. 7d. (**e**) pcDNA3-HA-Nix were transfected into HEK293T *Mavs*^−/−^ cells together with plasmids encoding Flag-tagged SUMO, MAVS, MAVS-(ΔCARD) or mini-MAVS as indicated. Thirty-six hours after transfection, cells were collected and subjected to immunoprecipitation. (**f**) HEK293T WT (Lane 1–5) and *Mavs-(M2-6L)* (Lane 6–11) cells were untreated, treated with 100 μM antioxidant PG for twelve hours, 1 μM rotenone for twelve hours, or transfected with constructs expressing sh-Nix or HA-Nix as indicated. Whole cell lysates were prepared for immunoblotting to determine the endogenous MAVS protein level. (**g**) HEK293T *Mavs*^*−/−*^ cells transfected with pcDNA3-flag-MAVS or pcDNA3-flag-MAVS-(W56R) were untreated, treated with 100 μM PG, 1 μM rotenone, 10 μM CCCP, or cotransfected with constructs expressing sh-Nix or HA-Nix as indicated. Whole cell lysates were prepared and subjected to immunoblotting.

Given that MAVS is located at mitochondria and prone to spontaneously aggregation in the absence of N-terminally truncated isoforms, we hypothesized that mitophagy was responsible for degradation of spontaneously-aggregated MAVS in *Mavs-(M2-6L)* cells. Indeed, total mitochondria as well as mitochondrial outer membrane protein VDCA1 and inner membrane protein cytochrome-c were dramatically decreased in *Mavs-(M2-6L)* cells compared to WT cells (Fig. 8a), indicating massive loss or downregulation of mitochondria. In addition, FACS analysis showed that both total mitochondria (stained with Mitotraker Green) and healthy mitochondria (stained with Mitotraker Red) were markedly reduced in *Mavs-(M2-6L)* cells (Fig. 8b). We next introduced the MAVS-W56R mutation into *Mavs-(M2-6L)* cells, which was reported to prevent MAVS filament formation and aggregation^15^. Strikingly, expression of the W56R mutant led to stabilization of MAVS in these cells. Other mitochondrial proteins were also restored in *Mavs-(M2-6L & W56R)* cells (Fig. 8a, Lane 4). In line with this, GFP-LC3 puncta was found only in *Mavs-(M2-6L)* but not WT, *Mavs-(W56R)* or *Mavs-(M2-6L & W56R)* cells (Supplementary Fig. 7c). As expected, MAVS cannot aggregate and produce IFN by treatment with sh-Beclin 1, sh-ATG5 or upon VSV infection in *Mavs-(M2-6L & W56R)* cells (Supplementary Fig. 7a,b).

We next determined which cargo receptor(s) might be required for recognition and mitophagic degradation of MAVS. By screening a panel of cargo receptors, we found that Nix and p62 interacted with wild type MAVS but not the aggregation-defective mutant MAVS-(W56R) (Fig. 8c). To test the functional roles of these cargo receptors, shRNAs targeting Nix, p62, PINK1, Parkin and MUL1 were introduced into *Mavs-(M2-6L)* cells alone or in combination. The results showed that IFN production and MAVS accumulation were detected only when Nix was knocked down (Fig. 8d and Supplementary Fig. 7e–g), indicating that degradation of aggregated MAVS is mediated by the Nix-dependent mitophagy instead of the PINK1/Parkin/MUL1-dependent pathway. In support of this result, mini-MAVS, a truncated MAVS harbouring only CARD domain and TM domain, could interact with Nix (Fig. 8e), while MAVS-(ΔCARD) cannot bind to Nix. Notably, mini-MAVS still interacted with Nix and was able to induce autophagy even after all lysines in the CARD domain were mutated (Supplementary Fig. 8a,b), demonstrating that recognition of aggregated MAVS by Nix is ubiquitin-independent.

It was reported that overexpressed MAVS led to mitochondrial membrane potential collapse, thus enhancing ROS production^20^,^41^. In line with this, transiently expressed MAVS and mini-MAVS induced ROS production and mitophagy in HEK293T *Mavs*^*−/−*^ cells (Supplementary Fig. 8c,d). We next examined if ROS and Nix corporately contributed to mitophagic degradation of spontaneously-aggregated MAVS. Induction of ROS by rotenone induced mitophagy and led to degradation of MAVS in both HEK293T WT and *Mavs-(M2-6L)* cells; while reduction of ROS by antioxidant propyl gallate (PG) only accumulated MAVS-(M2-6L) in *Mavs-(M2-6L)* cells (Fig. 8f), suggesting that spontaneously-aggregated MAVS was degraded by ROS-induced mitophagy. Although knock-down of Nix alone could stabilize MAVS-(M2-6L), we did not detect synergistic effect on MAVS-(M2-6L) accumulation by additional PG treatment. Furthermore, overexpression of Nix could not induce ROS production and mitophagy (Fig. 8f and Supplementary Fig. 8d). All of these results indicated that Nix is the downstream receptor of ROS-induced mitophagy that degrades MAVS aggregates. Moreover, PG and sh-Nix treatment led to the accumulation of wild type MAVS but not MAVS-(W56R) transiently expressed in HEK293T *Mavs*^*−/−*^ cells (Fig. 8g). Notably, knock-down of Nix blocked ROS-induced mitophagy and degradation of overexpressed MAVS by treatment with rotenone (Supplementary Fig. 8e), further establishing the essential role of Nix in ROS-induced mitophagic degradation of MAVS aggregates. Altogether, our data suggested that spontaneously-aggregated MAVS induced ROS production and mitophagy, which requires cargo receptor Nix for recognition and subsequent clearance of MAVS aggregates.

## Discussion

Ectopic overexpression of MAVS could induce IFN production independently of virus stimulation, by which MAVS was identified as an essential component in RIG-I pathway a decade ago. Therefore, it is tempting to speculate that there might be an inhibitor modulating the activity of endogenous MAVS in cells before stimulation. Up to date, many proteins have been reported to negatively regulate MAVS activity^38^,^42^,^43^,^44^,^45^. However, none of these proteins demonstrated a loss-of-function phenotype as such that MAVS would be activated spontaneously, indicating additional inhibitory mechanisms. Recent studies have revealed that MAVS activation is tightly coupled to its aggregation, which is mediated by its N-terminal CARD domain and induced by RIG-I-RNA complex^14^. In addition, recombinant sumo-MAVS-ΔTM was purified to contain two populations, including monomeric form and prion-like aggregated form, resembling endogenous MAVS functionally before and after viral infection respectively. Notably, monomeric sumo-MAVS-ΔTM could form prion-like aggregates spontaneously *in vitro*. Moreover, recombinant MAVS-CARD was identified predominantly in the filament form. Altogether, these results suggest that aggregation is an intrinsic propensity of recombinant MAVS, in contrast to endogenous MAVS which can only form aggregates following stimulation. In another words, there might be a mechanism preventing MAVS from spontaneous aggregation or activation in the absence of stimulus in cells. Our findings uncover that N-terminally truncated isoforms of MAVS mediate such a mechanism.

MAVS aggregation is mediated by its N-terminal CARD domain and therefore, N-terminally truncated isoforms of MAVS could not form aggregates by themselves. Instead, N-terminally truncated isoforms could associate with full-length MAVS through TM-mediated homotypic interaction, which may prevent spatially the proximity of neighboring CARDs and thus spontaneous aggregation of full-length MAVS. TMs from other membrane-bound proteins such as Bcl-xL, VAMP-2 and Pex13, could not interact with MAVS-TM or prevent MAVS aggregation, suggesting a very specific interaction between MAVS TMs. Following structural study might provide detailed mechanistic insight into such an interaction. Furthermore, we found that deletion of these truncated isoforms did not simply cause accumulation of aggregated full-length MAVS, but instead massive degradation of full-length MAVS. Our investigation further revealed that spontaneously-aggregated MAVS is promptly degraded by autophagy-mediated pathway. Indeed, autophagy is significantly induced in *Mavs-(M2-6L)* cells. Mounting evidence suggested that autophagy is a more selective process than originally anticipated. Selective autophagy of mitochondria or mitophagy is an important quality control mechanism that removes dysfunction mitochondria and aggregate-prone proteins. Our data showed that spontaneously-aggregated MAVS induced ROS in cells, which triggered Nix-mediated mitophagy for clearance of MAVS aggregates. Such a delicate mechanism could not only keep MAVS ready for transduction of an effective antiviral signalling, but also forestall the spontaneous activation of MAVS, which would lead to detrimental inflammation. Therefore, our finding might provide evidence showing the involvement of autophagy in protein quality control, which could be a general mechanism underlying disassembly of filamentous proteins in innate immune signalling^46^.

Filament formation is a common theme in innate immunity. For example, NLRP3, ASC and caspase-1, which are all core components in inflammasome pathway, function in a filament-dependent manner^16^,^46^. How these signalling molecules are kept from spontaneous aggregation in a quiescent cell and whether a similar mechanism to that mediated by MAVS truncated isoforms could be adopted remain to be explored. Nevertheless, many disorders, such as Huntington, Alzheimer and prion diseases^47^, were attributed to protein aggregation. While the precise pathological mechanisms underlying these diseases are still being debated, how relevant proteins are kept from spontaneous aggregation under physiological condition is poorly studied. Intriguingly, a naturally occurring C-terminal fragment of the prion protein (PrP) has been reported to delay disease progression and act as a dominant-negative inhibitor to PrP^Sc^ (the putative disease-causing form) formation with unknown mechanism^48^. Whether the C-terminal fragment of PrP could prevent PrP^Sc^ aggregation in a similar mechanism to N-terminally truncated isoforms of MAVS is worth following determination.

## Methods

### Plasmids and antibodies

Complementary DNA (cDNA) encoding MAVS ORF was amplified from HEK293T cells, which was fused to an N-terminal Flag tag and cloned into pcDNA3.0 vector between the restriction enzyme sites of XhoI and XbaI, namely pcDNA-flag-MAVS. Various isoforms and N-terminal truncations of MAVS were cloned into pcDNA3-flag vector^18^. pcDNA-flag-mini-MAVS was constructed by ligation of MAVS CARD domain (aa-1–100) to TM domain (aa-511–540). DNA fragments encoding MAVS-TM (aa-511–540), Bcl-xL-TM (aa-202–233), VAMP-2-TM (aa-84–116) and Pex13-TM (aa-136–233) were cloned into pcDNA3-flag-sumo^18^. pcDNA3-flag-MAVSΔ141-(Bcl-xL-TM) and pcDNA3-flag-MAVSΔ141-(VAMP-2-TM) were constructed by replacing MAVS-TM with Bcl-xL-TM and VAMP-2-TM respectively in vector pcDNA3-flag-MAVS-ΔN141. MAVS cDNA was cloned into pcDNA3 between restriction enzyme sites Hind III and Xba I, and mutants, including MAVS-(M2L), MAVS-(M2&6L), MAVS-(M2-5L) and MAVS-(M2-6L), were made by mutation of internal methionine to leucine as specified. pcDNA3-flag-Beclin 1 and pcDNA3-flag-ATG5 were made based on constructs from Dr Jiahuai Han (Xia’men University, China), and mutations were introduced to obtain shRNA-resistant forms. All mutations were made by using the Fast-mutagenesis Kit (Catalogue number FM111, TransGen Biotech, Beijing, China) or overlapping PCR strategy. Plasmids encoding various autophagy cargo receptors p62, Nix, NDP52, OPTN, NBR1 and TAX1BP1 were constructed by cloning of coding sequences from human complementary DNA into pcDNA3-HA vector. Primers are listed in Supplementary Table 1. All constructs were confirmed by DNA sequencing. Antibodies anti-MAVS-(N), anti-MAVS-(M) and anti-MAVS-(C) were raised by immunizing rabbits with recombinant proteins His-sumo-hMAVS-(aa-1–100), His-sumo-hMAVS-(aa-301–460) or His-sumo-hMAVS-(aa-460–510), respectively. Commercial antibodies included anti-tubulin (Sigma, T5168, dilution 1:7,500), anti-prohibitin (Abcam, ab75766, dilution 1:10,000), anti-p53 (Cell Signaling Technology, 3724S, dilution 1:1,000), anti-Beclin 1 (Santa Cruz Biotechnology, sc-11,427, dilution 1:1,000), anti-ATG5 (Cell Signaling Technology, 12994S, dilution 1:1,000), anti-LC3 (Sigma, L8198, dilution 1:3,000) anti-IRF3 (Abcam, 2241-1, dilution 1:3,000), anti-p62 (Sigma, p0067, dilution 1:1,000), anti-Nix (Abcam, ab8399, dilution 1:1,000), anti-Flag (Sigma, F3165, F7425, dilution 1:5,000), anti-Flag M2 (FITC) (Sigma, F4049, dilution 1:500 for immunofluorescence), anti-HA (Cell Signaling Technology, 3724S, dilution 1:2,000).

### Cells and viruses

HEK293T (originally from Dr Zhijian ‘James’ Chen at University of Texas Southwestern Medical Center at Dallas, USA) and HeLa cells were cultured in Dulbecco’s modified Eagle’s medium (DMEM) medium supplemented with 10% fetal bovine serum (FBS, ExCell Bio, FSP500), penicillin (100 U ml^−1^) and streptomycin (100 μg ml^−1^). A549 cells were grown in F-12 K Nutrient Mixture medium with 10% FBS and antibiotics. THP-1 cells were cultured in RPMI Medium 1,640 basic medium with 10% FBS and antibiotics. HeLa (TCHu187), A549 (TCHu150) and THP-1 (TCHu57) cells were from the Cell Resource Center (Shanghai Institute of Biochemistry and Cell Biology, Chinese Academy of Sciences). MG132 (S2619), Bortezomib (S1013), 3-MA (S2767), LY294002 (S1105) and chloroquine (S4430) were purchased from Selleckchem. Rotenone (R8875), CCCP (C2759) and antioxidant PG (48710) were purchased from Sigma. Sendai virus (Cantell strain) from Charles River Laboratories was amplified in house and used at a concentration of 50 HA units/ml. Recombinant virus VSV-ΔM51-GFP was propagated in vero cells and used with a multiplicity of infection (MOI) of 1.0 (ref. 18).

### Generation of knock-in cell lines in HEK293T

For generation of HEK293T *Mavs-(M2L)* cell line, TALEN-mediated genomic knock-in was employed. Briefly, two TALEN constructs was generated using the FastTALE^TM^TALEN Assembly Kit (Catalogue number 2,801, Sidansai Biotechnology, Shanghai, China), to create a double-stranded break at the fourth exon of the human *Mavs* gene encoding the region covering Methinonine142. A donor plasmid, which contained sequences flanking the fourth exon and harboured a mutation for changing Methionine142 to Leucine, was constructed for homologous recombination. Two TALEN constructs and the donor plasmid were transfected into HEK293T cells using Lipofectamine 2,000 (Invitrogen). Individual cellular colonies were picked up 2 weeks after transfection and genomic DNAs were then extracted and sequenced to verify modification on the *MAVS* gene. HEK293T *Mavs-(M2&6L)*, *Mavs-(M2-5L)* and *Mavs-(M2-6L)* cell lines were generated using the same strategy, which encoded MAVS with mutations of M303L, M358L, M367L and M449L as indicated. HEK293T *Mavs-(W56R) and Mavs-(M2-6L & W56R)* cell lines were generated based on HEK293T and *Mavs-(M2-6L)* cell lines respectively, by replacing Tryptophan56 with Arginine in *Mavs* gene. Oligonucleotides and TALEN-targeting sequences were listed in Supplementary Table 1.

### Subcellular fractionation and IRF3 dimerization assay

Subcellular fractionation was performed as described^18^. Briefly, cells were homogenized in hypotonic buffer (10 mM Tris-Cl (pH 7.5), 10 mM KCl, 0.5 mM EGTA and 1.5 mM MgCl_2_), and the homogenates were then centrifuged at 1,000 g for 5 min to isolate the supernatant (S1). Subsequently, S1 was centrifuged at 10,000 g for 10 min to separate the supernatant (S5) and pellet (P5). S5 was further concentrated at 4 °C, 100,000 g for 30 min, and the resultant supernatant (S100) was collected. P5 fractions containing the crude mitochondria were used for IRF3 dimerization assay *in vitro*, as described in ref. 18. Sucrose gradient centrifugation was performed, as described in ref. 14.

### Immunoprecipitations

HEK293T cells were transfected with indicated constructs. Thirty-six hours post-transfection, cells were washed with cold PBS buffer and resuspended with lysis buffer (HEPES 20 mM (pH 7.5), NaCl 150 mM, Triton X-100 1%, DTT 1 mM and PMSF 1 mM). Following a brief concentration, cellular lysate was incubated with anti-flag M2 affinity gel (Sigma A2220) at 4 °C for 4 h. M2 beads were then spun down and washed for three times with lysis buffer. IP products were directly subjected to SDS-PAGE and immunoblotting.

### Fluorescence microscopy

Various expression vectors as specified were transfected into HeLa cells by Lipofectmine 2000. After 36 h of transfection, cells were incubated with 150 nM Mitrotracker Red CMXRos (Thermo Fisher Scientific) for 15 min at room temperature. After staining, cells were washed with pre-warmed 1 × PBS and fixed with 4% formaldehyde for 15 min. Cells were then permeabilized with 0.5% TritonX-100 for 15 min, and incubated with anti-Flag M2 (FITC) for overnight. To examine VSV proliferation, cells were infected with VSV-DM51-GFP. Eight hours later, cells were taken for observation of fluorescence intensity. All the images were taken by Olympus IX71 inverted fluorescence microscope.

### Flow cytometric analyses

Mitochondrial mass was measured by fluorescence levels upon staining with Mitrotracker Green FM at 200 nM and Mitrotracker Red CMXRos at 150 nM (Thermo Fisher Scientific) for 20 min at 37 °C. Mitochondria associated ROS levels were measured using the ROS assay kit (Catalogue number C1300, APPLYGENE, Beijing, China) according to manufacturer’s instructions. Cells were then washed with PBS, trypsinized and resuspended in PBS for FACS analysis. FACS data were collected using BD LSR II Flow Cytometer (BD Biosciences, New Jersey, USA) and analysed by FlowJo version 7.6.1.

### Luciferase reporter assay

The procedure was as described. Briefly, HEK293T cells (1.0 × 10^5^ per well) were seeded into 12-well plates and co-transfected with 20 ng of IFN-luciferase as reporter gene, 20 ng of pCMV-LacZ as internal control, and indicated expression vectors by the calcium phosphate method. After 36 h of post-transfection, cells were lysed in Passive Lysis Buffer (Promega) and firefly luciferase activity was assessed via luminometer using the Luciferase Reporter Kit (Catalogue number E1910, Promega). LacZ activities were measured by ONPG assay following a protocol provided by Sigma Technical Bulletin (GALA-1KT). Fold induction of firefly luciferase was normalized to LacZ activity. Data were expressed as fold induction over empty vector-transfected controls.

### Quantitative PCR

Total RNA from cells was extracted with RNA simple total RNA kit (Catalogue number DP419, Tiangen, Shanghai, China) and reversed-transcribed using the GoScript Reverse Transcription system (Promega). cDNAs were then used as templates for qPCR assay using SuperReal Premix Plus (Tiangen). Data were normalized by the level of internal control GAPDH expression in each individual sample. The 2^−ΔΔCt^ method was used to calculate relative expression changes. qPCR primers are listed in Supplementary Table 1.

### Treatment with shRNA

Vectors encoding shRNAs were constructed using pLKO.1. Sequences of shRNAs targeting Beclin 1 and ATG5 can be found in Supplementary Table 1. In brief, HEK293T WT and *Mavs-(M2-6L)* cells were seeded into 6-well plates or 10-cm dishes and transfected with indicated shRNA-encoded vectors using Lipofectamine 2,000 (Invitrogen). After 36 h of post transfection, cells were subjected for the second transfection. Cells were collected at 36 h after the second transfection for the following assays.

### Semidenaturing detergent agarose gel electrophoresis

Crude mitochondria (P5) were isolated from HEK293T cells, resuspended in the sample buffer (0.5 × TBE, 10% glycerol, 2% SDS and 0.0025% bromophenol blue) and loaded onto a vertical 1.5% agarose gel. After electrophoresis in the running buffer (1 × TBE and 0.1% SDS) for 30 min with a constant voltage of 100 V at 4 °C, western immunoblotting was performed.

### Plaque assay

Cells were infected with VSV at MOI=1 and aliquots of culture media were taken at 12 h. HEK293T cells in 6-well plates were infected with serial dilutions of the recovered viruses for 1 h. Cells were overlaid with 1% soft agar in DMEM and incubated for 48 h. Plates were stained with 0.1% crystal violet in DMEM to display plaques, which were then quantitated.

### Statistical analysis

All data are presented as mean values ±s.d. on the basis of three independent experiments. Statistical significance between groups was determined by unpaired two-tailed Student’s *t*-test. Differences were considered to be significant when *P*<0.05.

### Data availability

The data that support the findings of this study are available from the corresponding author on reasonable request.

## Additional information

**How to cite this article:** Qi, N. *et al*. Multiple truncated isoforms of MAVS prevent its spontaneous aggregation in antiviral innate immune signalling. *Nat. Commun.* **8,** 15676 doi: 10.1038/ncomms15676 (2017).

**Publisher’s note:** Springer Nature remains neutral with regard to jurisdictional claims in published maps and institutional affiliations.

## Supplementary Material

Supplementary Information Supplementary Figures and Supplementary Table 1

Peer Review File
