# Supplementary material for: Multiple truncated isoforms of MAVS prevent its spontaneous aggregation in antiviral innate immune signalling
Source: Nat Commun. 2017 Jun 13;8:15676. doi: 10.1038/ncomms15676 (PMC5474743; doi:10.1038/ncomms15676)
Supplement: Supplementary Information — Supplementary Figures and Supplementary Table 1 [file ncomms15676-s1.pdf]

Supplementary Figure 1

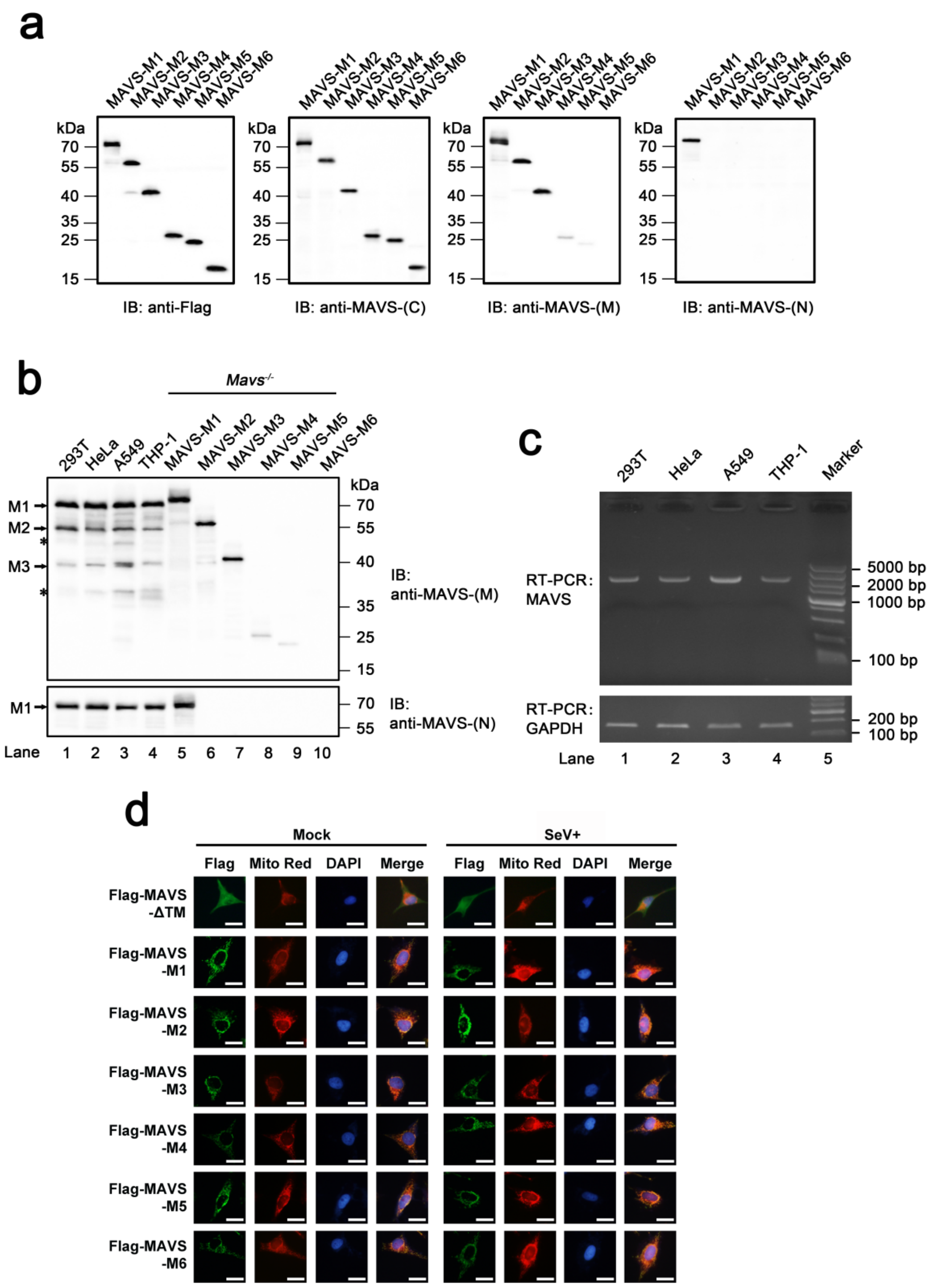

Supplementary Figure 1 │ Characterization of six MAVS isoforms.

(a) Immunoblotting to show the migration position of Flag-tagged MAVS isoforms. HEK293T *Mavs*$^{-/-}$ cells were transfected with constructs expressing

Flag-tagged MAVS-M1/M2/M3/M4/M5/M6. Whole cell lysates were subjected to immunoblotting with anti-Flag, anti-MAVS-(C), anti-MAVS-(M) and anti-MAVS-(N) antibodies, respectively.

(b) Immunoblotting to show endogenous MAVS isoforms in various human cell lines with anti-MAVS-(M) and anti-MAVS-(N) antibodies. Samples were the same as those used in Fig. 1c.

(c) Reverse transcribed-PCR (RT-PCR) showing *Mavs* transcripts from various human cell lines. Total RNAs were extracted from HEK293T, HeLa, A549 and THP-1 cells. cDNA was prepared and subjected to RT-PCR using primers targeting *Mavs* mRNA 5'-UTR and 3'-UTR. GAPDH was analyzed as an internal control.

(d) Plasmids encoding Flag-tagged MAVS-M1/M2/M3/M4/M5/M6 and MAVS-ΔTM were transfected into HeLa cells respectively. Twenty-four hours after transfection, cells were uninfected or infected with Sendai virus. Fluorescent images were taken twelve hours after infection and immunofluorescence staining. Anti-Flag M2 (FITC) was used for immunofluorescence staining of Flag-tagged proteins. Mitochondria were stained with Mitrotracker Red. Nucleus was stained with DAPI. Scale bar represents 5 micrometers.

Supplementary Figure 2

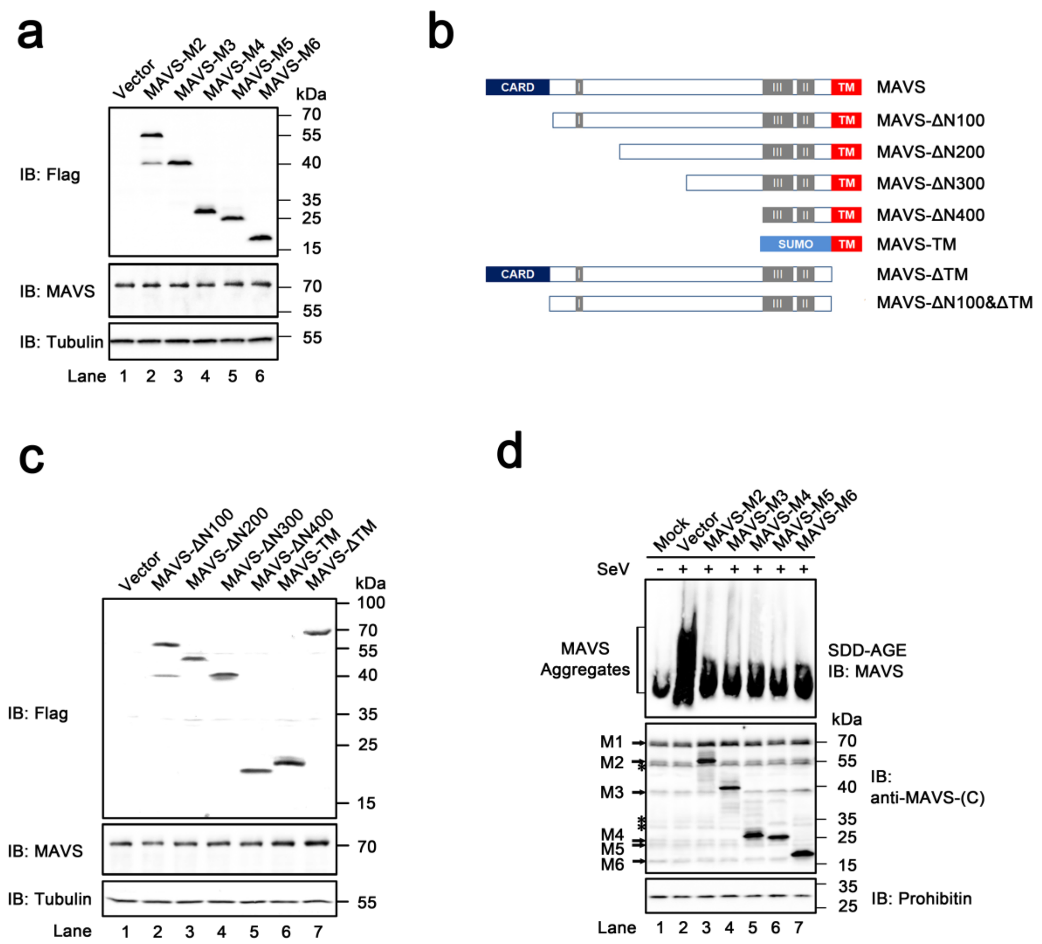

Supplementary Figure 2 │ Expression level of MAVS isoforms and a series of N-terminally truncated MAVS (related to Fig. 2) in HEK 293T cells.

(a) Immunoblotting to show the protein levels of transiently expressed MAVS isoforms (M2-M6) and endogenous MAVS in HEK293T cells after Sendai virus infection as indicated in Fig. 2a.

(b) A diagram illustrating various truncated forms of MAVS, including deletions of N-terminal 100 aa, 200aa, 300aa, 400aa, 510aa (i.e., MAVS-TM), or C-terminal 30aa (i.e., MAVS-ΔTM).

(c) Immunoblotting to show the protein levels of transiently expressed N-terminally truncated MAVS and endogenous MAVS in HEK293T cells after Sendai virus infection as indicated in Fig. 2b.

(d) Transiently expressed N-terminally truncated MAVS isoforms inhibited endogenous MAVS aggregation under viral stimulation. pcDNA3-flag vector or pcDNA3-flag-MAVS-M2/M3/M4/M5/M6 was transfected into HEK293T cells respectively. The cells were infected with Sendai virus twenty-four hours after transfection. Cells were harvested twelve hours post virus infection, which were subjected to subcellular fractionation to get P5 and S5 fraction. P5 fractions were used to examine MAVS aggregation. Immunoblotting to show the protein levels of transiently expressed MAVS isoforms (M2-M6) and endogenous MAVS with anti-MAVS-(C) antibody.

Supplementary Figure 3

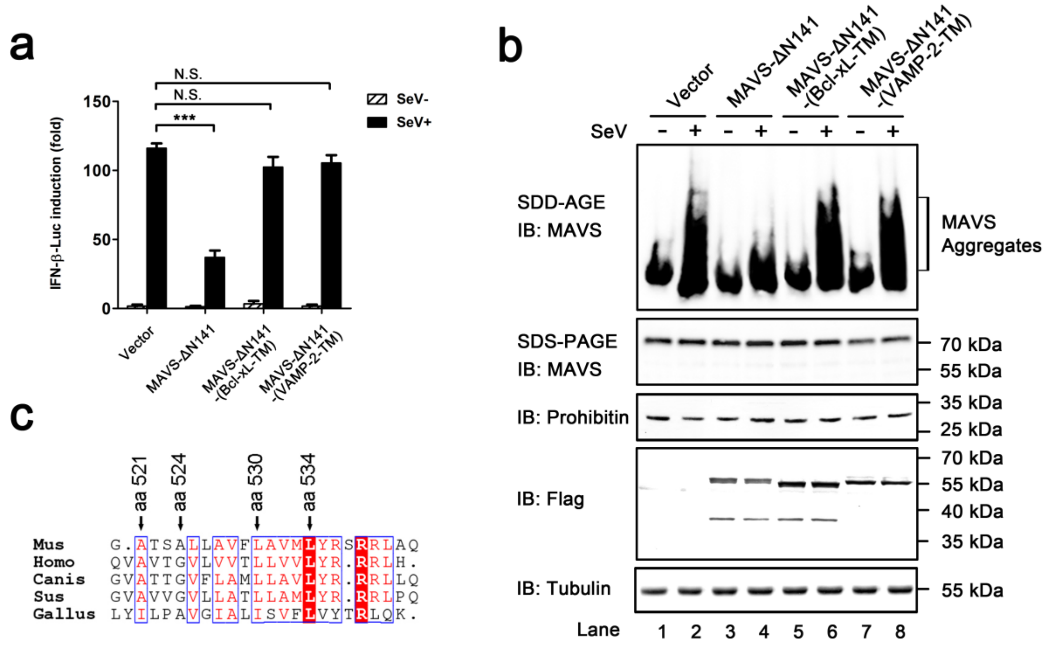

Supplementary Figure 3 │ N-terminally truncated forms of MAVS inhibit full-length MAVS activity and aggregation through its TM domain.

(a, b) Experiments were performed as described in Fig. 3c and 3d respectively except that pcDNA3-flag-MAVS-ΔN141, pcDNA3-flag-MAVS-ΔN141-(Bcl-xL-TM),pcDNA3-flag-MAVS-ΔN141-(VAMP-2-TM) were transfected into HEK293T cells. To determine the endogenous MAVS protein level and avoid signal from overexpressed MAVS mutants, anti-MAVS-(N) antibody was used for immunoblotting. All data are presented as the mean values based on three independent experiments, and error bars indicate s.d. *P* values were determined by unpaired two-tailed Student's t-test. ****P*<0.001. N.S. indicates no statistically significant difference.

(c) A diagram showing the highly conservative amino acids in TM domains of

MAVS among various species. Sequences of MAVS TM domains from indicated species were subjected to alignment. Highly conservative amino acids were labeled with red character and indicated as arrows.

Supplementary Figure 4

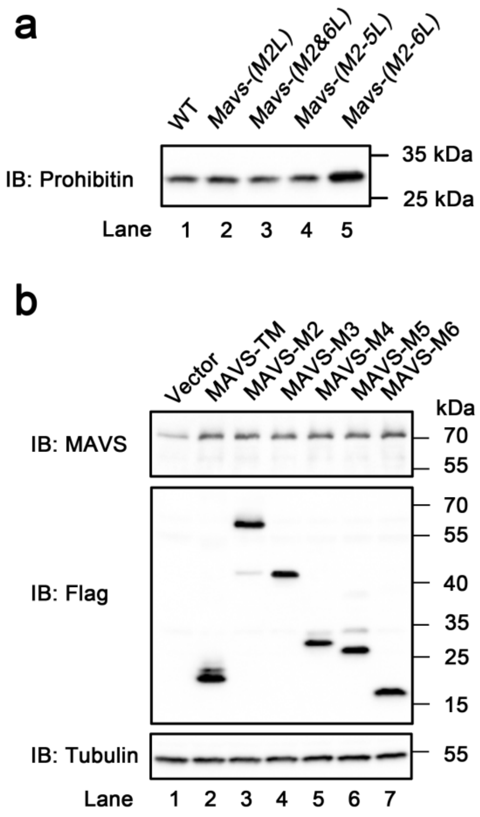

Supplementary Figure 4 │ N-terminally truncated MAVS isoforms stabilized MAVS in *Mavs-(M2-6L)* cells.

(a) Immunoblotting of internal control Prohibitin to show the relative protein level of endogenous MAVS in various cell lines as indicated. Samples were the same as those used in Fig. 5a.

(b) HEK293T *Mavs-(M2-6L)* cells were transfected with empty vector or plasmids encoding MAVS TM domain and various N-terminally truncated isoforms as indicated. Transfection was performed as described in Fig. 5f. Whole cell lysates were obtained to determine the endogenous MAVS protein level by immunoblotting.

Supplementary Figure 5

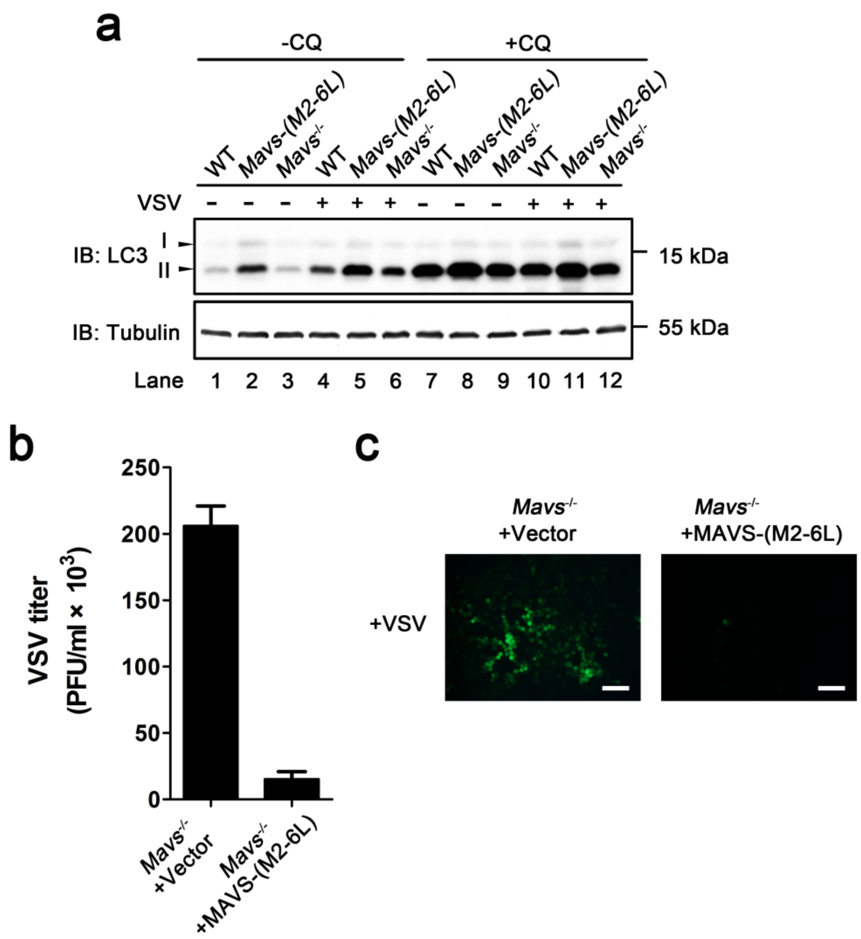

Supplementary Figure 5 │ Infection with VSV changed the relative ratio of autophagy marker LC3-II/-I in various HEK293T cell lines as indicated.

(a) HEK293T WT, *Mavs*$^{-/-}$ and *Mavs-(M2-6L)* cells were uninfected or infected with VSV (MOI=1). At twelve hours post infection, cells were untreated or treated with chloroquine (CQ) for four hours. Whole cell lysates were prepared for immunoblotting of autophagy marker LC3.

(b, c) As described in Fig. 6g, HEK293T *Mavs*$^{-/-}$ cells transfected with empty vector or plasmid expressing MAVS-(M2-6L) were infected with VSV (MOI=1).

At twelve hours post infection, VSV titers were quantitated by plaque assay (b). Fluorescent images were taken to examine VSV proliferation at eight hours after infection (c). Scale bar represents 10 micrometers. All data are presented as the mean values based on three independent experiments, and error bars indicate s.d.

Supplementary Figure 6

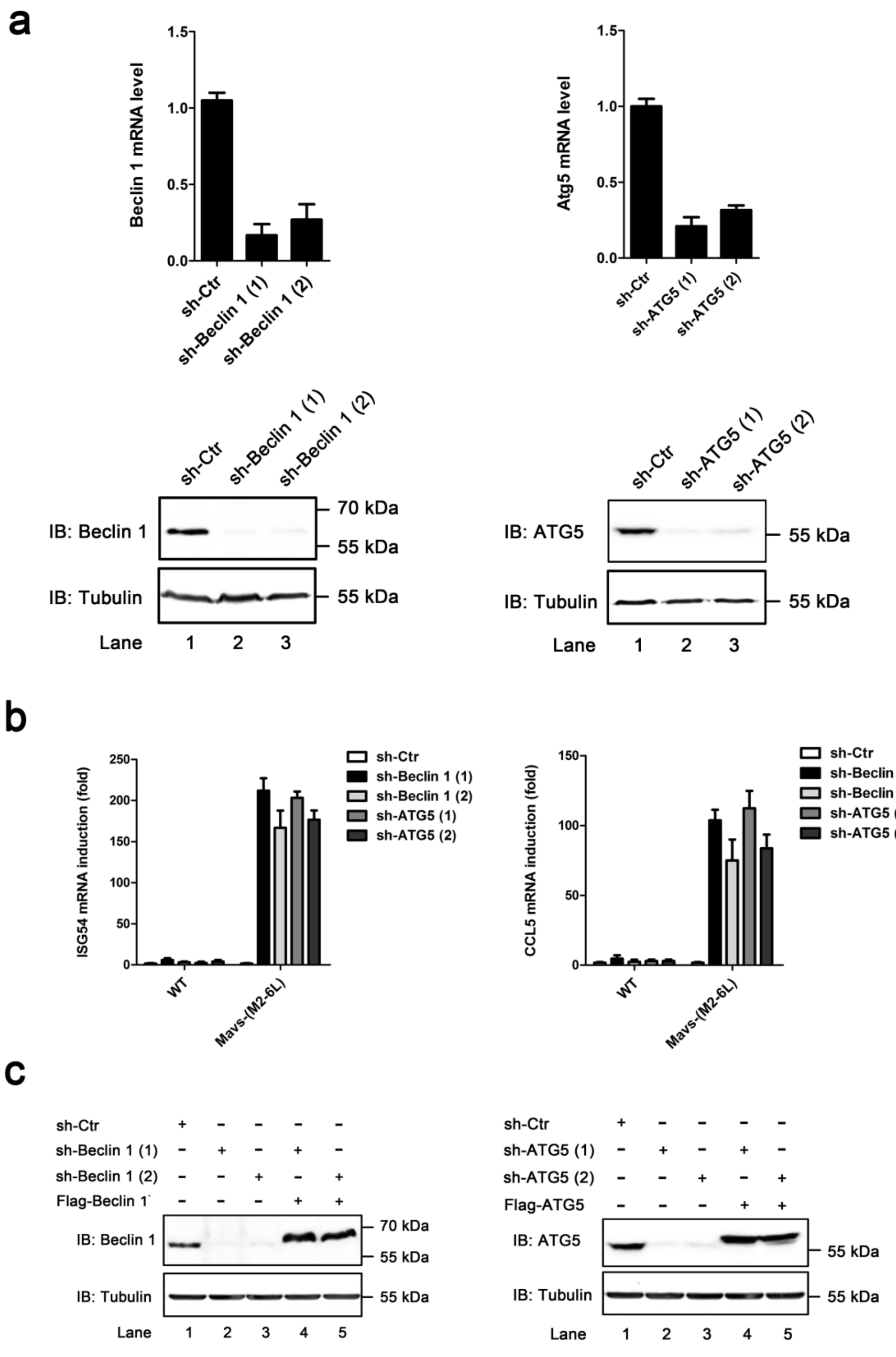

Supplementary Figure 6 │ mRNA and protein levels of Beclin 1 and ATG5 in HEK293T *Mavs-(M2-6L)* cells, related to Fig. 7.

(a) HEK293T *Mavs-(M2-6L)* cells were treated with shRNAs as described in

Fig. 7a. qPCR and immunoblotting to show the knock-down efficiency of Beclin 1 and ATG5 by indicated shRNAs at mRNA level and protein level respectively. All data are presented as the mean values based on three independent experiments, and error bars indicate s.d.

(b) Experiments were performed as described in Fig. 7b. ISG54 and CCL5 inductions were then measured by qPCR.

(c) Experiments were performed as described in Fig. 7c,d. Immunoblotting to show the protein levels of endogenous Beclin 1 and ATG5 as well as transiently expressed Flag-Beclin 1 and Flag-ATG5.

Supplementary Figure 7

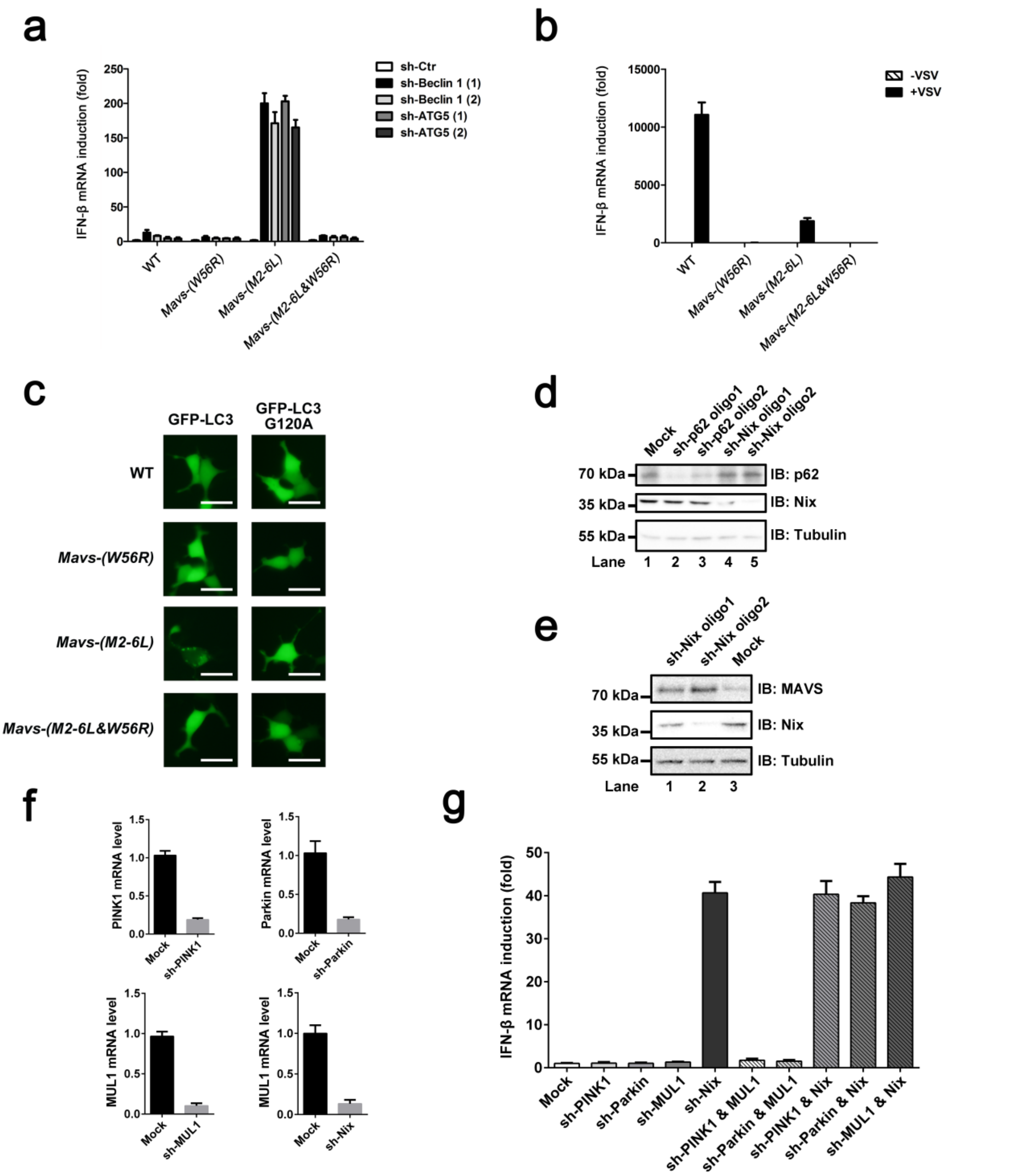

Supplementary Figure 7 │ Characterization of HEK293T WT, *Mavs-(W56R)*, *Mavs-(M2-6L), Mavs-(M2-6L&W56R)* cell lines (related to Figure 8a) and knock-down efficiency of shRNAs (related to Figure 8d).

(a) As described in Fig. 7a, HEK293T WT, *Mavs-(W56R)*, *Mavs-(M2-6L)* and *Mavs-(M2-6L&W56R)* cell lines were treated with shRNAs targeting Beclin 1 or

ATG5. RNA was extracted and qPCR was performed to measure IFN induction. All data are presented as the mean values based on three independent experiments, and error bars indicate s.d.

(b) HEK293T WT, *Mavs-(W56R)*, *Mavs-(M2-6L)* and *Mavs-(M2-6L&W56R)* cell lines were infected with VSV at MOI=1 for twelve hours. RNA was extracted from harvested cells and qPCR was performed to measure IFN induction. All data are presented as the mean values based on three independent experiments, and error bars indicate s.d.

(c) HEK293T WT, *Mavs-(W56R)*, *Mavs-(M2-6L)* and *Mavs-(M2-6L&W56R)* cell lines were transfected with plasmids encoding GFP-tagged LC3 or its loss-function mutant G120A as expression control. Fluorescent images were taken at thirty-six hours after transfection. Scale bar represents 5 micrometers.

(d) Immunoblotting to examine the knock-down efficiency of various shRNA oligoes targeting p62 or Nix used in Fig. 8d.

(e) As described in Fig. 8d, HEK293T *Mavs-(M2-6L)* cells were transfected with constructs encoding shRNA oligo1 and oligo2 targeting Nix. Whole cell lysates were prepared for immunoblotting to determine the endogenous MAVS and Nix protein level.

(f) qPCR to examine the knock-down efficiency of various shRNA oligoes targeting the indicated mitophagy related genes.

(g) Constructs encoding shRNAs targeting various mitophagy related genes were transfected into *Mavs-(M2-6L)* cells alone or in combination as indicated.

Transfection was performed as described in Fig. 8d. RNA was extracted from harvested cells and qPCR was performed to measure IFN induction. All data are presented as the mean values based on three independent experiments, and error bars indicate s.d.

Supplementary Figure 8

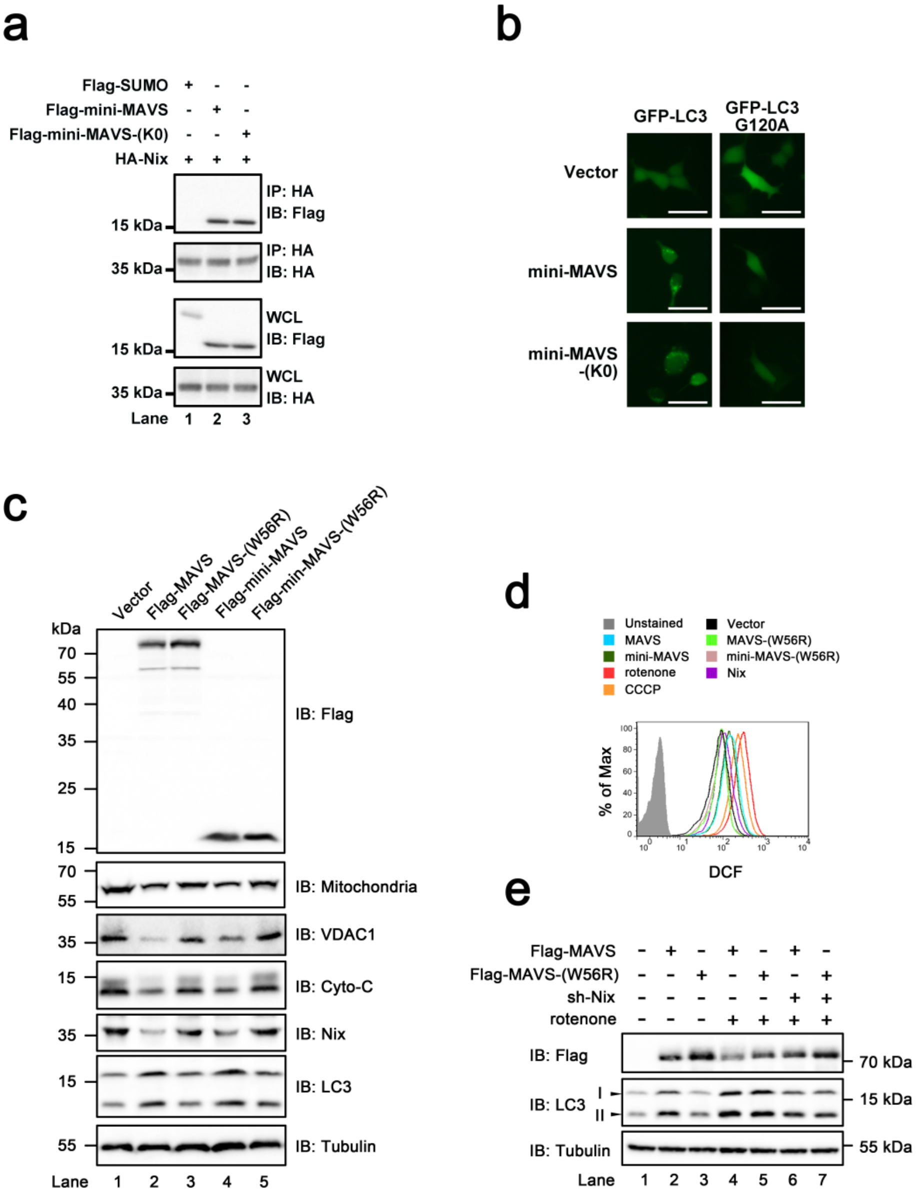

Supplementary Figure 8 │ Transiently expressed MAVS and mini-MAVS in HEK293T *Mavs*$^{-/-}$ cells induced mitophagy through ROS production.

(a) pcDNA3-HA-Nix was transfected into HEK293T *Mavs*$^{-/-}$ cells together with plasmids encoding Flag-tagged SUMO, mini-MAVS or mini-MAVS-(K0). Thirty-six hours after transfection, cells were harvested and subjected to immunoprecipitation.

(b) Empty vector, pcDNA3-flag-mini-MAVS or pcDNA3-flag-mini-MAVS (K0) were transfected into HEK293T *Mavs*$^{-/-}$ cells, together with plasmids encoding GFP-tagged LC3 or its loss-function mutant G120A as expression control. Fluorescent images were taken at thirty-six hours after transfection. Scale bar represents 5 micrometers.

(c) HEK293T *Mavs*$^{-/-}$ cells were transfected with empty vector, pcDNA3-flag-MAVS, pcDNA3-flag-MAVS-(W56R), pcDNA3-flag-mini-MAVS, or pcDNA3-flag-mini-MAVS-(W56R). Thirty-six hours after transfection, whole cell lysates were prepared for SDS-PAGE. Immunoblotting was performed using antibodies as indicated.

(d) As described in Fig. 8b, HEK293T *Mavs*$^{-/-}$ cells transfected with empty vector, pcDNA3-flag-MAVS, pcDNA3-flag-MAVS-(W56R), pcDNA3-flag-mini-MAVS, pcDNA3-flag-mini-MAVS-(W56R) and pcDNA3-HA-Nix were collected for FACS analysis to determine ROS production. Rotenone and CCCP treatment was analyzed as positive control. Histograms of FACS analysis are depicted. FACS gating strategy can be found in Supplementary Fig. 10.

(e) As described in Fig. 8g, HEK293T *Mavs*$^{-/-}$ cells transfected with pcDNA3-flag-MAVS or pcDNA3-flag-MAVS-(W56R) were untreated, treated with 1 μM rotenone, or cotransfected with constructs encoding sh-Nix as indicated. Whole cell lysates were prepared and subjected to immunoblotting.

Supplementary Figure 9

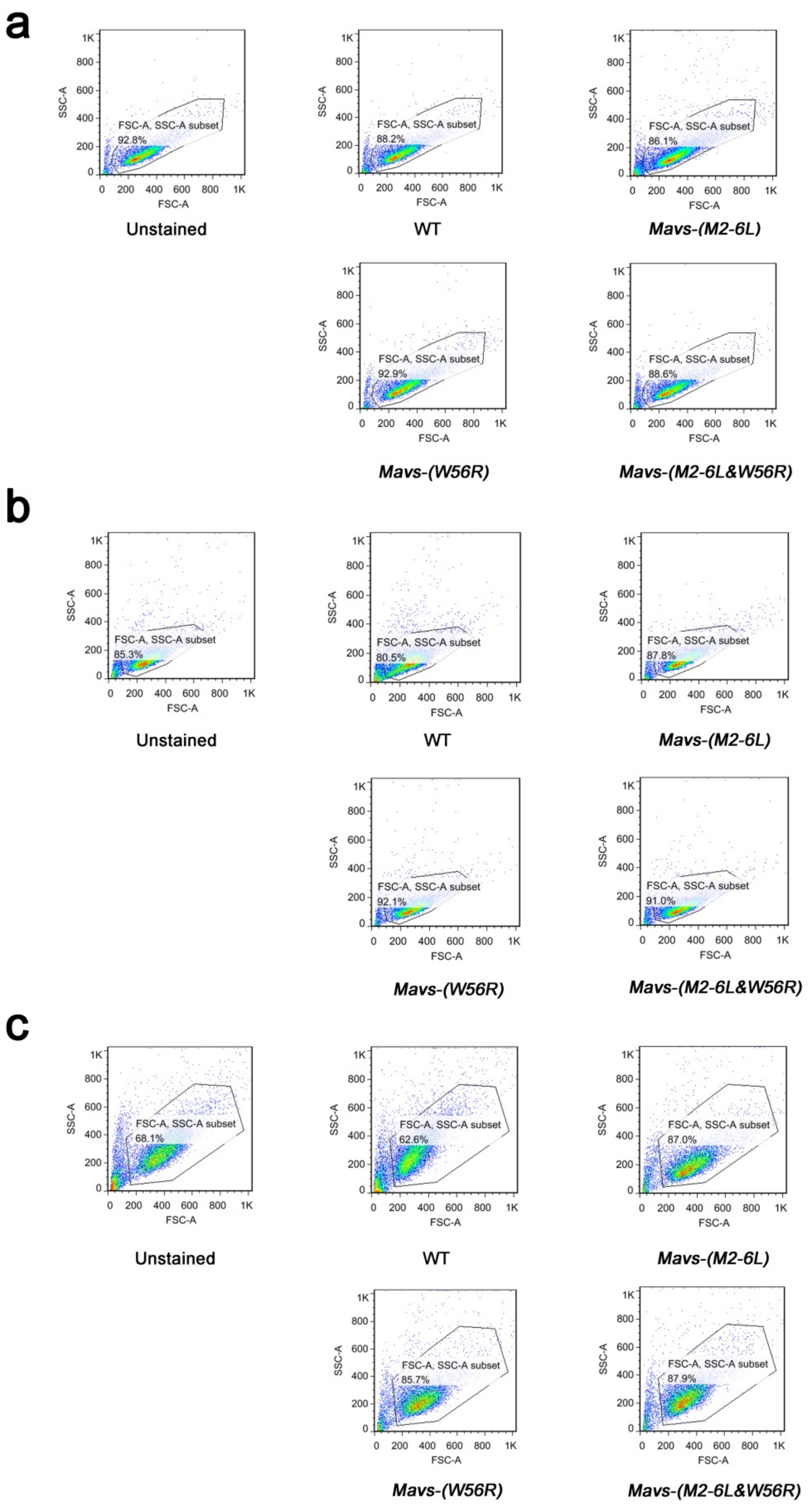

Supplementary Figure 9 │ Gating strategy for FACS data shown in Fig. 8b.

Cell lines stained with various dyes were gated according to their FSC (forward scatter) and SSC (sideward scatter), as well as green/red fluorescent features. (a) Mitotracker green; (b) Mitotracker red; (c) DCF.

Supplementary Figure 10

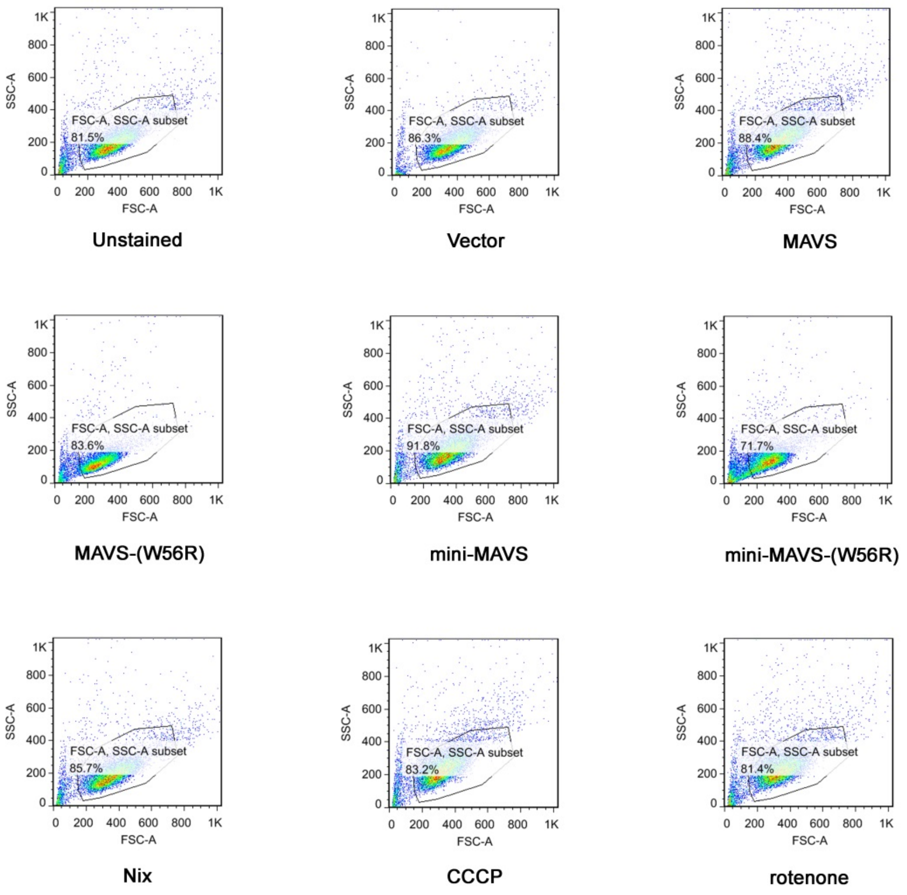

Supplementary Figure 10 │Gating strategy for FACS data shown in Supplementary Fig. 8d.

Transfected or treated HEK293T *Mavs*$^{-/-}$ cells were stained with DCF and gated according to their FSC (forward scatter) and SSC (sideward scatter), as well as green fluorescent features.

Supplementary Figure 11

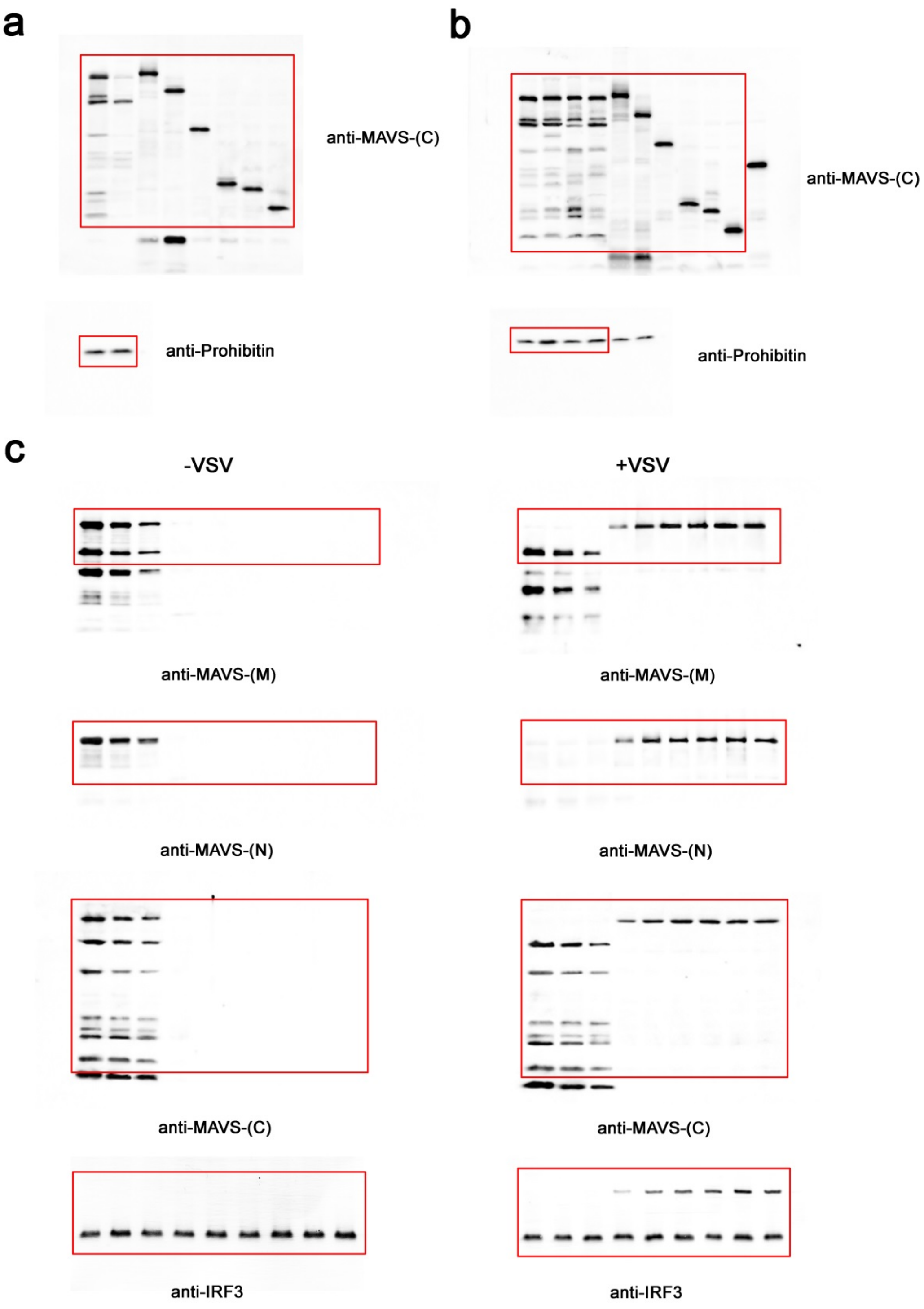

Supplementary Figure 11 │Full blot images.

(a) For Fig. 1b. (b) For Fig. 1c. (c) For Fig. 1d.

Supplementary Figure 12

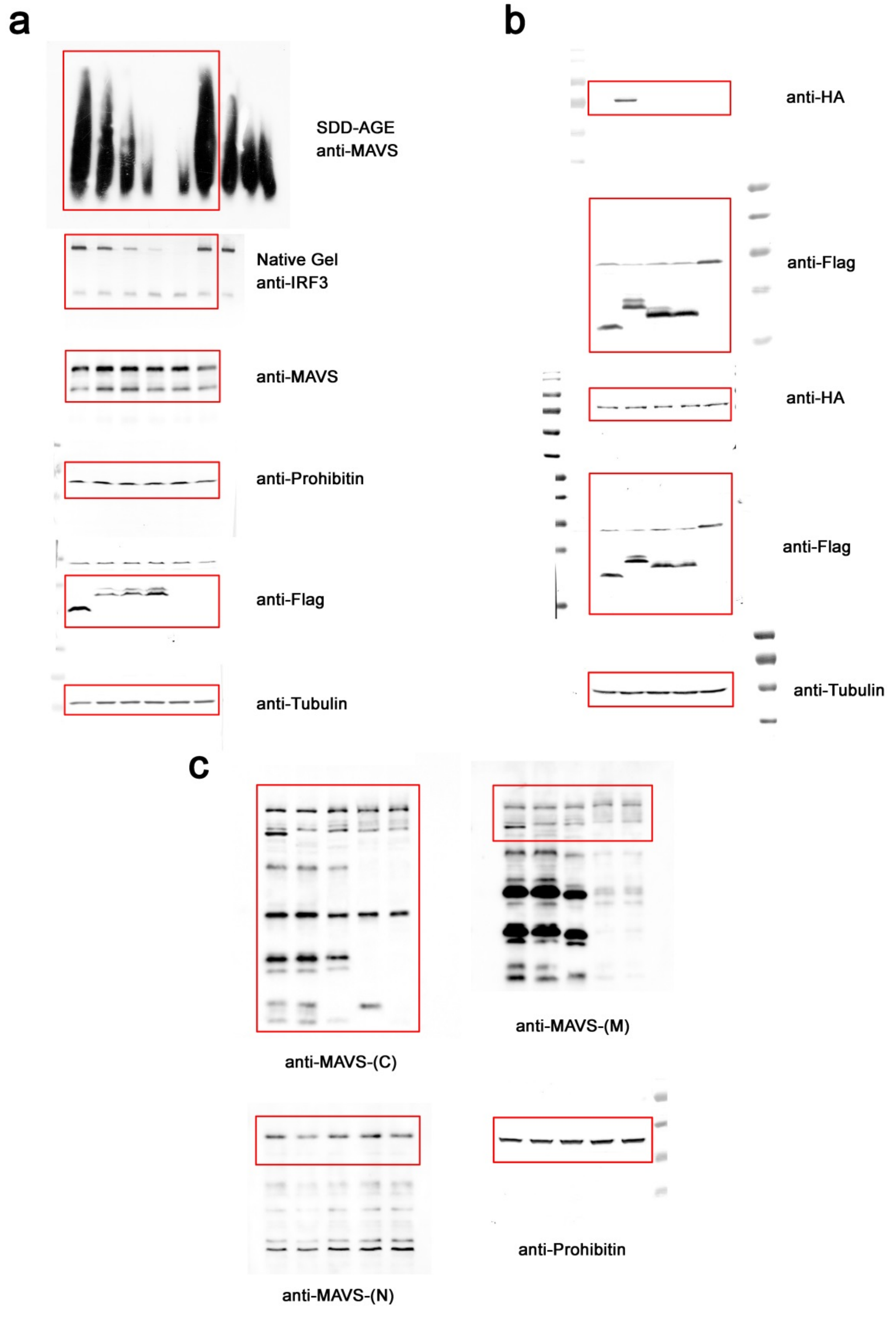

Supplementary Figure 12 │Full blot images.

(a) For Fig. 2c. (b) For Fig. 3b. (c) For Fig. 4b.

Supplementary Figure 13

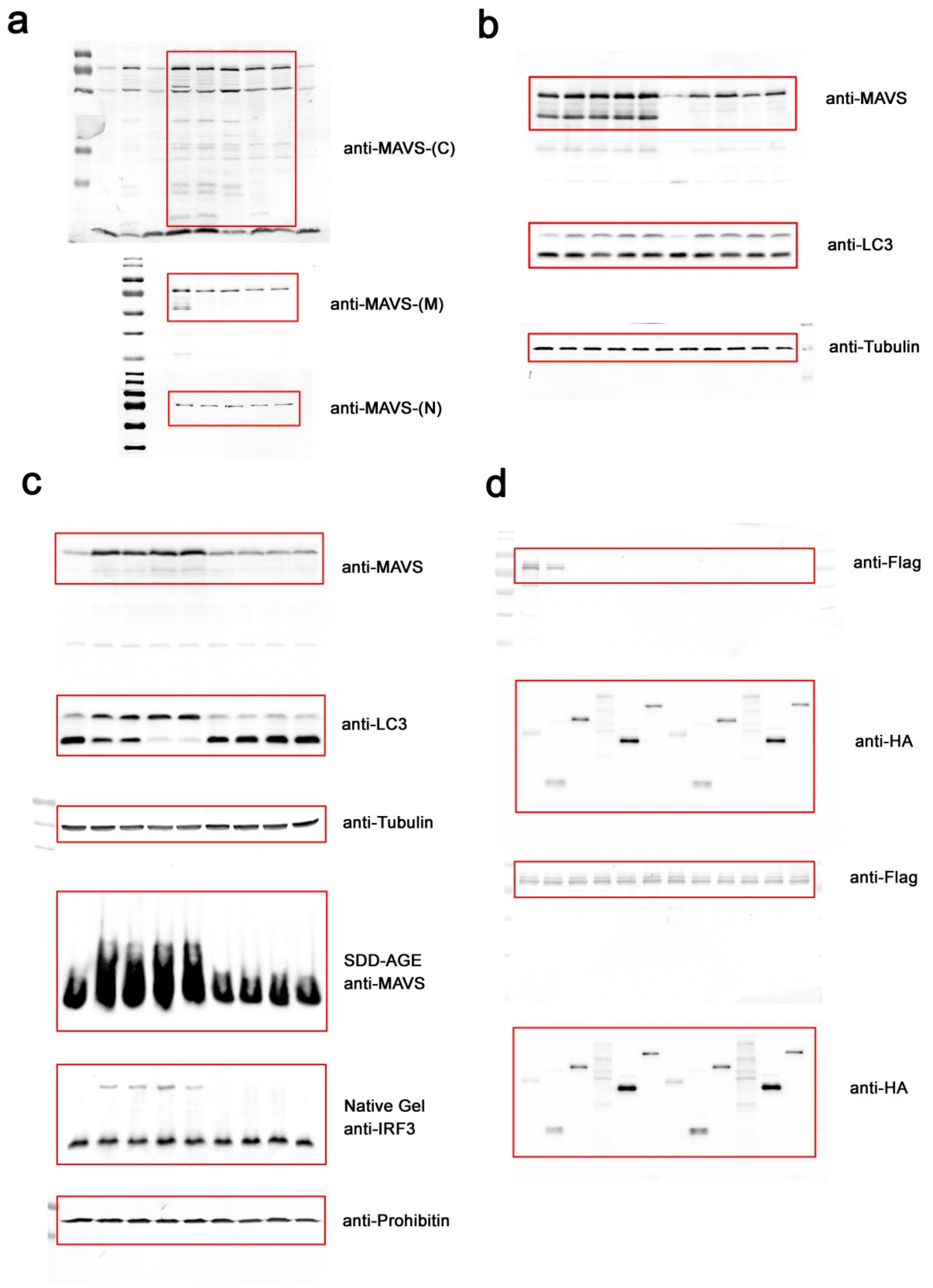

Supplementary Figure 13 │Full blot images.

(a) For Fig. 5a. (b) For Fig. 6b. (c) For Fig. 7d. (d) For Fig. 8c.

Supplementary Table 1 Sequence of primers for molecular cloning and TALEN-mediated gene editing or gene knockdown.

Gene ID / Name	Sequence (5' to 3')	Purpose
MAVS-M1-For	AAACTCGAGATGCCGTTTGCTGAAGACAAGACCT	Protein expression
MAVS-M2-For	AAACTCGAGATGCCTGTCCAGGAGACCCAGGCGC	
MAVS-M3-For	AAACTCGAGATGCCTGTGAACACAGTGGCCCTGA	
MAVS-M4-For	AAACTCGAGATGGTGCCATCCAAAGTGCCTACTAG	
MAVS-M5-For	AAACTCGAGATGGTGCTCACCAAGGTGTCTG	
MAVS-M6-For	AAACTCGAGATGGGGCCCTGCCATGGCCCAGA	
MAVS-ΔN100-For	AAACTCGAGGACCGTCCCCCAGACCCACTG	
MAVS-ΔN200-For	AAACTCGAGGAACTGGGCAGTACCCACAC	
MAVS-ΔN300-For	AAACTCGAGACCTTGATGCCTGTGAACAC	
MAVS-ΔN400-For	AAACTCGAGAGCTCAGCCTGGCTAGACAG	
MAVS-TM-For	AAACTCGAGCCCTCACCTGGGGCTCTGTG	
MAVS-Rev	AAATCTAGACTAGTGCAGACGCCGCCGGTAC	
MAVS-ΔTM-For	AAACTCGAGATGCCGTTTGCTGAAGACAAGAC	
MAVS-ΔTM-Rev	AAATCTAGATTACCTGTGGCATGGCACCTCCC	
Bcl-xL-TM-Rev-For1	GGGAGGTGCCATGCCACAGGGAGAGTCGAAAGGGC CAGG	
Bcl-xL-TM-Rev-For2	AAACTCGAGGAGAGTCGAAAGGGCCAGG	
Bcl-xL-TM-Rev	AAATCTAGATCATTTCCGACTGAAGAGTG	
VAMP-2-TM-For1	GGGAGGTGCCATGCCACAGGGCTCAAGCGCAAATA CTGGTG	
VAMP-2-TM-For2	AAACTCGAGGCTCAAGCGCAAATACTGGTG	
VAMP-2-TM-Rev	AAATCTAGATTAAGAGCTGAAGTAAACTATG	
Pex13-TM-For	AAACTCGAGATTGAGCATGCATTTGCCTCTGACA	
Pex13-TM-Rev	AAATCTAGATTAAGATTTTGCTGAGGTAGCTGCT	
MAVS-M1-For	CCCAAGCTTATGCCGTTTGCTGAAGACAAGAC	
MAVS-M2L-For	CCCTGCCTGTCCAGGAGACCCAGGCGCCAGAGTCC CCAGGAGAGA	
MAVS-M2L-Rev	GGTCTCCTGGACAGGCAGGGGGTAACTTGGCTCCT TCTCTCTGCA	
MAVS-M3L-For	CCACCTTGCTGCCTGTGAACACAGTGGCCCTGAAAG TGCCTGCC	
MAVS-M3L-Rev	GTTCACAGGCAGCAAGGTGGTAGGCACTTTGGAGG GCAGAGAG	
MAVS-M4, 5L-For	GCCTGGTGCCATCCAAAGTGCCTACTAGCCTGGTGC TCACCAAGGTGTCTGCCAG	
MAVS-M4, 5L-Rev	CCAGGCTAGTAGGCACTTTGGATGGCACCAGGCCA GCACGGGTTGAGTTGATG	
MAVS-M6L-For	GGCCTGGGGCCCTGCCATGGCCCAGAGGAGAATGA GTATAAGT	
MAVS-M6L-Rev	CCATGGCAGGGCCCCAGGCCCAAGGAGGTGCTGGC	

	ACTGATGGCAAGA	
MAVS-TM-A521W-For	GGGCTCTGTGGCTCCAGGTGTGGGTGACAGGGGTG CTGGTAGTC	
MAVS-TM-A521W-Rev	CCACACCTGGAGCCACAGAGC	
MAVS-TM-G524W-For	GGCTCCAGGTGGCTGTGACATGGGTGCTGGTAGTC ACACTCCTGG	
MAVS-TM-G524W-Rev	CCATGTCACAGCCACCTGGAG	
MAVS-TM-L530W-For	CAGGGGTGCTGGTAGTCACATGGCTGGTGGTGCTG TACCGGCG	
MAVS-TM-L530W-Rev	CCATGTGACTACCAGCACCCC	
MAVS-TM-L534W-For	GGTAGTCACACTCCTGGTGGTGTGGTACCGGCGGC GTCTGCACTAG	
MAVS-TM-L534W-Rev	CCACACCACCAGGAGTGTGACTAC	
MAVS-W56R-For	CCGGCATCTCTTCAATACCCTTCAGCGGCGGCCCGG CTGGGT	
MAVS-W56-Rev	GAAGGGTATTGAAGAGATGCCGGAGGGTGTCCCGG TTCCCTGAGA	
Beclin 1-For	AAAGGATCCATGGAAGGGTCTAAGACGTCCAAC	
Beclin 1-Rev	TATGCGGCCGCTCATTTGTTATAAAATTGTGAGGACA CCC	
Beclin 1-Resistant-For1	AACTCTGGAGAAGAGCCTTTTATTGAAACTCCTCGCC AGGATGGTG	
Beclin 1-Resistant-Rev1	AATAAAAGGCTCTTCTCCAGAGTTAGTCTCTTCCTCC TGGGTCTCTCCT	
Beclin 1-Resistant-For2	AAGATCGAGGATACTGGTGGCAGTGGCGGCTCCTAT TCCATCAAA	
Beclin 1-Resistant-Rev2	CACTGCCACCAGTATCCTCGATCTTGCCTTTCTCCAC ATCCATCCTG	
ATG5-For	AAACTCGAGATGACAGATGACAAAGATGTGCTTCG	
ATG5-Rev	AAATCTAGATCAATCTGTTGGCTGTGGGATGATA	
ATG5-Resistant-For1	AACACCTTAGCTATCCGGACAATTTTCTTCATATTAGT ATCATCCCACAGCCA	
ATG5-Resistant-Rev1	AAATTGTCCGGATAGCTAAGGTGTTCACTCAGCCACT GCAGAGGTGTTT	
ATG5-Resistant-For2	CACAAGCAGCTGTGGATGGGTTTGCAAAATGACAGA TTTGACCAGT	
ATG5-Resistant-Rev2	TTTGCAAACCCATCCACAGCTGCTTGTGATCTTTTTT CTGCATTTCA	
P62-For	AAACTCGAGATGGCGTCGCTCACCGTGAAGGCC	
P62-Rev	AAATCTAGATTACAACGGCGGGGGATGCTTTGAATAC	
Nix-For	AAACTCGAGATGTCGTCCCACCTAGTCGAGCC	
Nix-Rev	AAATCTAGATTAGTAGGTGCTGGCAGAGGGTGTGC	
NDP52-For	TTTGGATCCATGGAGGAGACCATCAAAGATC	

NDP52-Rev	TTTGCGGCCGCTTAGAGAGAGTGGCAGAACACG	
OPTN-For	AAACTCGAGATGTCCCATCAACCTCTCAGCTGC	
OPTN-Rev	AAATCTAGATTAAATGATGCAATCCATCACGTG	
TAX1BP1-For	TTTGGATCCATGACATCCTTTCAAGAAGTCC	
TAX1BP1-Rev	TTTGCGGCCGCTTAGTCAAAATTTAGAACATTCTG	
NBR1-For	AAACTCGAGATGGAACCACAGGTTACTCTAAATG	
NBR1-Rev	AAATCTAGATTAGAACCAGGAGAATGCTTCAC	
Mavs-M2L-TALEN Left arm	ACAACAGCTGCAGAGAGAA	TALEN-mediated knock-in cell line
Mavs-M2L-TALEN Right arm	GGGTCTCCTGGACAGGCAT	
Mavs-M2L-HR-For1	ACCTCCTAATCCACCTGCCT	
Mavs-M2L-HR-Rev1	ATTCCCAGACCCTCTGTCCA	
Mavs-M2L-HR-For2	CCTGGACAGGCAGTGGGTAACTTGGCTCCTTCTC	
Mavs-M2L-HR-Rev2	GTTACCCACTGCCTGTCCAGGAGACCCA	
Mavs-M6L-TALEN Left arm	CAGTGCCAGCACCTCCT	
Mavs-M6L-TALEN Right arm	CATTCTCCTCTGGGCCAT	
Mavs-M6L-HR-For1	GGTACCTTCAAAAGTGCCTACTAGTCTGGTGCTCAC	
Mavs-M6L-HR-Rev1	TCTGCCTCTAAAGGAGTAGCCAAGA	
Mavs-M6L-HR-For2	CAGCACCTCCTTAGGACTGGGGCCCTG	
Mavs-M6L-HR-Rev2	CAGTCCTAAGGAGGTGCTGGCA	
Mavs-M3-5L-TALEN Left arm	GCCTGCCAACCCAGCATCT	
Mavs-M3-5L-TALEN Right arm	TTGAGCTAGTTGGCAACTT	
Mavs-M3-5L-HR-For1	ATGTGCATTCCGAGTTCCGT	
Mavs-M3-5L-HR-Rev1	AGCCTCCACAATCCCCAAGAAA	
Mavs-M3-5L-HR-For2	CCTACCACCTTGCTGCCTGTGAACACAGTG	
Mavs-M3-5L-HR-Rev2	GTTCACAGGCAGCAAGGTGGTAGGCACTTT	
Mavs-M3-5L-HR-For2	GGTACCTTCAAAAGTGCCTACTAGTCTGGTGCTCAC	
Mavs-M3-5L-HR-Rev2	TAGTAGGCACTTTTGAAGGTACCAGGCCAGCACGG	
Mavs-W56R-TALEN Left arm	CGGCGGCCCGGCTGGGT	
Mavs-W56R-TALEN Right arm	GCTCACAGCCCCTCAGT	
Mavs-W56R-HR-For	CAGCTTCCTTGTTTGTTATTGTCGG	
Mavs-W56R-HR-Rev	TCAAACTCAATCTCATCAAATCGCC	
si-MAVS-sense	CCACCUUGAUGCCUGUGAA	siRNA and shRNA oligoes
si-MAVS-antisense	UUCACAGGCAUCAAGGUGG	
sh-Beclin 1 (1)-For	CCGGAACTCAGGAGAGGAGCCATTTCTCGAGAAATGGCTCCTCTCCTGAGTTTTTTTG	
sh-Beclin 1 (1)-Rev	AATTCAAAAAAACTCAGGAGAGGAGCCATTTCTCGAGAAATGGCTCCTCTCCTGAGTT	
sh-Beclin 1 (2)-For	CCGGAAGATTGAAGACACAGGAGGCCTCGAGGCCTCCTGTGTCTTCAATCTTTTTTTG	

sh-Beclin 1 (2)-Rev	AATTCAAAAAAAGATTGAAGACACAGGAGGCCTCGAGGCCTCCTGTGTCTTCAATCTT	
sh-ATG5 (1)-For	CCGGAAGCAACTCTGGATGGGATTGCTCGAGCAATCCCATCCAGAGTTGCTTTTTTTG	
sh-ATG5 (1)-Rev	AATTCAAAAAAAGCAACTCTGGATGGGATTGCTCGAGCAATCCCATCCAGAGTTGCTT	
sh-ATG5 (2)-For	CCGGAACATCTGAGCTACCCGGATACTCGAGTATCCGGGTAGCTCAGATGTTTTTTTG	
sh-ATG5 (2)-Rev	AATTCAAAAAAACATCTGAGCTACCCGGATACTCGAGTATCCGGGTAGCTCAGATGTT	
sh-Ctr-For	CCGGAATTCTCCGAACGTGTCACGTCTCGAGACGTGACACGTTCGGAGAATTTTTTTG	
sh-Ctr-Rev	AATTCAAAAAAATTCTCCGAACGTGTCACGTCTCGAGACGTGACACGTTCGGAGAATT	
sh-P62 (1)-For	CCGGAAGGATGACATCTTCCGAATCCTCGAGGATTCGGAAGATGTCATCCTTTTTTTG	
sh-P62 (1)-Rev	AATTCAAAAAAAGGATGACATCTTCCGAATCCTCGAGGATTCGGAAGATGTCATCCTT	
sh-P62 (2)-For	CCGGAACAGATGGAGTCGGATAACTCTCGAGAGTTATCCGACTCCATCTGTTTTTTTG	
sh-P62(2)-Rev	AATTCAAAAAAACAGATGGAGTCGGATAACTCTCGAGAGTTATCCGACTCCATCTGTT	
sh-Nix (1)-For	CCGGAAGACATGGAGAAGATTCTTTCTCGAGAAAGAATCTTCTCCATGTCTTTTTTTG	
sh-Nix (1)-Rev	AATTCAAAAAAAGACATGGAGAAGATTCTTTCTCGAGAAAGAATCTTCTCCATGTCTT	
sh-Nix (2)-For	CCGGAAGTCGAGGCTTTGAAGAAAACTCGAGTTTTCTTCAAAGCCTCGACTTTTTTTG	
sh-Nix (2)-Rev	AATTCAAAAAAAGTCGAGGCTTTGAAGAAAACTCGAGTTTTCTTCAAAGCCTCGACTT	
sh-PINK1-For	CCGGAAGCCATCTTGAACACAATGACTCGAGTCATTGTGTTCAAGATGGCTTTTTTTG	
sh-PINK1-Rev	AATTCAAAAAAAGCCATCTTGAACACAATGACTCGAGTCATTGTGTTCAAGATGGCTT	
sh-Parkin-For	CCGGAAGGAGGTGGTTGCTAAGCGACTCGAGTCGCTTAGCAACCACCTCCTTTTTTTG	
sh-Parkin-Rev	AATTCAAAAAAAGGAGGTGGTTGCTAAGCGACTCGAGTCGCTTAGCAACCACCTCCTT	
sh-MUL1-For	CCGGAAGGAGCTGTGCGGTCTGTTACTCGAGTAACAGACCGCACAGCTCCTTTTTTTG	
sh-MUL1-Rev	AATTCAAAAAAAGGAGCTGTGCGGTCTGTTACTCGAGTAACAGACCGCACAGCTCCTT	
Mavs-5'-UTR-For	GGTACCCGAGTCTCGTTTCC	

Mavs-3'-UTR-For	AGCCAAGGCATGTCCTGCT	qRT-PCR
IFN-β-For	CAGCAGTTCCAGAAGGAGGA	
IFN-β-Rev	AGCCAGGAGGTTCTCAACAA	
GAPDH-For	AGAAGGCTGGGGCTCATTTG	
GAPDH-Rev	AGGGGCCATCCACAGTCTTC	
CXCL10-For	TGGCATTCAAGGAGTACCTC	
CXCL10-Rev	TTGTAGCAATGATCTCAACACG	
ISG54-For	CTGAACCGAGCCCTGCCGAAC	
ISG54-Rev	GCTGCCTCGTTTTGCCCTTTGAG	
CCL5-For	ATCCTCATTGCTACTGCCCTC	
CCL5-Rev	GCCACTGGTGTAGAAATACTCC	
qBeclin 1-For	TGTCACCATCCAGGAACTCA	
qBeclin 1-Rev	CCTGGCGAGGAGTTTCAATA	
qATG5-For	GCTATTGATCCTGAAGATGGG	
qAtg5-Rev	GATGTTCACTCAGCCACTG	
qNix-For	CCGCCCCTGCACAACAACAAC	
qNix-Rev	GCCTCTGGAACTACTCTGTCC	
qPINK1-For	CGGCCTGCAGCTGGGTCGAGC	
qPINK1-Rev	GCCCGCACCACGAACTGCCG	
qParkin-For	GGGTTCCGGCTGACCAGTTGC	
qParkin-Rev	GCAGAATGACAGCCAGCCCC	
qMUL1-For	CACCGCCGCCCTGTACTCCG	
qMUL1-Rev	TCCTGAAGTGTCAGCCGCTG	
