# Supplementary material for: Multiple truncated isoforms of MAVS prevent its spontaneous aggregation in antiviral innate immune signalling
Source: Nat Commun. 2017 Jun 13;8:15676. doi: 10.1038/ncomms15676 (PMC5474743; doi:10.1038/ncomms15676)
Supplement: Peer Review File [file ncomms15676-s2.pdf]

Editorial Note: this manuscript has been previously reviewed at another journal that is not operating a transparent peer review scheme. This document only contains reviewer comments and rebuttal letters for versions considered at Nature Communications.

REVIEWERS' COMMENTS:

Reviewer #1 (Remarks to the Author):

This is a technically sound study that demonstrates that multiple short isoforms of MAVS contribute as negative regulators to prevent activation/association of full-length MAVS. The data are consistent with previous reports and add more information by identifying multiple MAVS isoforms and by demonstrating a role of mitophagy. The article will be of interest to specialists in the field of pathogen sensing.
